# A HOPS Protein, MoVps41, Is Crucially Important for Vacuolar Morphogenesis, Vegetative Growth, Reproduction and Virulence in *Magnaporthe oryzae*

**DOI:** 10.3389/fpls.2017.01091

**Published:** 2017-06-30

**Authors:** Xiaojie Zhang, Guanghui Wang, Chengdong Yang, Jun Huang, Xiaofeng Chen, Jie Zhou, Guangpu Li, Justice Norvienyeku, Zonghua Wang

**Affiliations:** ^1^State Key Laboratory for Ecological Pest Control of Fujian and Taiwan Crops and College of Life Science, Fujian Agriculture and Forestry UniversityFuzhou, China; ^2^Fujian University Key Laboratory for Functional Genomics of Plant Fungal Pathogens, Fujian Agriculture and Forestry UniversityFuzhou, China; ^3^Department of Biochemistry and Molecular Biology, University of Oklahoma Health Sciences Center, Oklahoma CityOK, United States; ^4^College of Ocean Science, Minjiang UniversityFuzhou, China

**Keywords:** MoVps41, HOPS complex, *Magnaporthe oryzae*, homotypic-vacuolar fusion, pathogenesis

## Abstract

The homotypic fusion and protein sorting protein complex (HOPS) is the first known tether complex identified in the endocytic system that plays a key role in promoting homotypic vacuolar fusion, vacuolar biogenesis and trafficking in a wide range of organisms, including plant and fungi. However, the exact influence of the HOPS complex on growth, reproduction and pathogenicity of the economically destructive rice blast fungus has not been investigated. In this study, we identified *M. oryzae* vacuolar protein sorting 41 (MoVps41) an accessory subunit of HOPS complex and used targeted gene deletion approach to evaluate its contribution to growth, reproduction and infectious life cycle of the rice blast fungus. Corresponding results obtained from this study showed that MoVps41 is required for optimum vegetative development of *M. oryzae* and observed that MoVps41 deletion mutant displayed defective vegetative growth. Our investigation further showed that MoVps41 deletion triggered vacuolar fragmentation, compromised membrane integrity and pathogenesis of the *ΔMovps41* mutant. Our studies also showed for the first time that MoVps41 plays an essential role in the regulation of sexual and asexual reproduction of *M. oryzae.* In summary, our study provides insight into how MoVps41 mediated vacuolar fusion and biogenesis influences reproduction, pathogenesis, and vacuolar integrity in *M. oryzae* and also underscores the need to holistically investigate the HOPS complex in rice blast pathogen.

## Introduction

Rice blast disease caused by filamentous ascomycete fungus *Magnaporthe oryzae* has been regarded as the most devastating disease limiting rice cultivation worldwide (Talbot, 2003; Dean et al., 2012). Studies have shown that the initiation of plant infection involves the successful germination of viable conidia landing on potentially susceptible host tissues and the subsequent differentiation of germ tubes into a functional infectious structure called appressoria, which glues itself firmly to the host tissues (Nesher et al., 2008; Wetherbee et al., 2012). The matured appressoria develops robust penetration peg and generate enormous turgor that enables it to physically puncture and invade host cells resulting in the manifestation of the typical dark-brown diamond-shaped blast lesions on leaves, culm, neck, panicles, and roots (Kankanala et al., 2007; Talbot and Wilson, 2009; Marcel et al., 2010). Previous comparative genomic studies conducted with genome assembled sequence of the rice blast fungus has shown that the *M. oryzae* genome contains numerous effector proteins (Yoshida et al., 2016) and subsequent functional genetic studies have revealed that the aserminal model organism deploys these effectors during host-pathogen interaction to suppress host immunity for successful establishment of blast disease (Dou and Zhou, 2012; Mentlak et al., 2012; Yan and Talbot, 2016; Zheng et al., 2016). Although most of the effector proteins identified in the rice blast fungus does not possess identifiable secretion peptide (Soanes et al., 2007; Petre and Kamoun, 2014), experimental evidence, however, showed that the successful delivery or export of these effector proteins into the cytoplasm of host cells are largely mediated by the vacuoles (Chaudhari et al., 2014).

Vacuoles are ubiquitously present and extremely complex cellular organelles found in all eukaryotes and some prokaryotes; vacuoles play essential roles in nutrient storage, degradation of macromolecules, ion homeostasis, and regulation of pH, autophagy, lysis and recycling of misfolded proteins (Matile, 1978; Klionsky et al., 1990; Li and Kane, 2009; Richards et al., 2012). Research findings have shown that, several independent trafficking machineries mediate the successful transportation of misfolded proteins, other cargoes and proteins earmarked for secretion to the vacuoles in both plants and fungi (Pereira et al., 2014). Furthermore, vacuoles promote growth, efficient cell differentiation, strengthen symbiotic interactions and enhance the pathogenesis of filamentous fungus (Soanes et al., 2008; Pollack et al., 2009). In addition to sorting of misfolded proteins, the vacuolar complex also serves as a site for several other important cellular activities, including biogenesis, tethering, docking and fusion of cargoes and membrane proteins for transport (Hammer and Sellers, 2012; Barlowe and Miller, 2013). More so, the HOPS complex functions as a tether at vacuoles for different membrane and organelles including late endosomes, AP-3 transport vesicles and autophagosomes (Schröter et al., 2016; Spang, 2016), the HOPS complex acting as tether interacts with SNAREs and/or GTPases to control the specificity of vesicle fusion in various organisms (Janková Drdová, 2017).

The activities of SNARE proteins are largely responsible for the docking of vesicles with an organelle and evidence currently available showed that *S. cerevisiae*, SNARE proteins play roles beyond the facilitation of protein transport by promoting homotypic vacuolar fusion processes (Yu and Hughson, 2010; Ariosa and Klionsky, 2016). Generally, SNARE associated homotypic fusion processes are mainly regulated by homotypic fusion and protein sorting complex (HOPS) (Hong and Lev, 2014), according to Chen (2016), the HOPS complex principally consists of core subunit (Vps11, Vps16, Vps18, and Vps33) and an accessory subunit (Vps39 and Vps41). Previous research demonstrations have shown that Vps39 and Vps41 inactivity impacted negatively on vacuolar morphology and aggregation (Raymond et al., 1992; Guerra and Bucci, 2016; Medvedev et al., 2016), furthermore, the accessory subunit of the HOPS complex are crucially involved in the promotion of autophagy vegetative growth, conidiation and virulence of many filamentous fungi (Liu et al., 2006; Yang et al., 2016). Previous research findings have shown that MoVps39 a component of the HOPS complex crucially regulate conidiogenesis, appressorium formation, and development of blast infection in *M. oryzae.*

However, the intrinsic role of HOPS complex proteins have not been extensively studied in the cosmopolitan rice blast fungus, subsequently we identified and used functional genetic tools to explore the contributions of MoVps41 in fungal development, pathogenicity, and vacuolar biogenesis.

## Results

### Identification of MoVps41

To obtain the homolog of Vps41 sequence from *M. oryzae* genome database, we used the amino acid sequence of budding yeast to conduct BLASTp search through the now defunct Magnaporthe genome resource^1^ and successfully identified Vps41 homologous protein (1357-amino acid) encoded by MGG_03313.7 (MoVps41) and proceeded to perform domain prediction with http://prosite.expasy.org/. Our domain prediction analysis identified two domains motifs namely; the clathrin heavy chain repeat (CHCR) domain and the RING-H2 motif (Supplementary Figure S1). Further sequence alignment analysis showed that MoVps41 shares high sequence homology with the Vps41 proteins from *Saccharomyces cerevisiae, Fusarium graminearum, Aspergillus nidulans, Arabidopsis thaliana, Mus musculus*, and *Homo sapiens* (Supplementary Figure S2). The CHCR domain is highly conserved among all the organisms analyzed, with 38 to 72% identity between *M. oryzae* and mammals, plants and fungi (Supplementary Table S2), whilst the RING-H2 motif appeared absent in the Vps41 sequences from *Saccharomyces cerevisiae* and *A. nidulans* (Supplementary Figure S1A and Table S2).

### *MoVPS41* Is Required for Vegetative Development of *M. oryzae*

**FIGURE 1:**
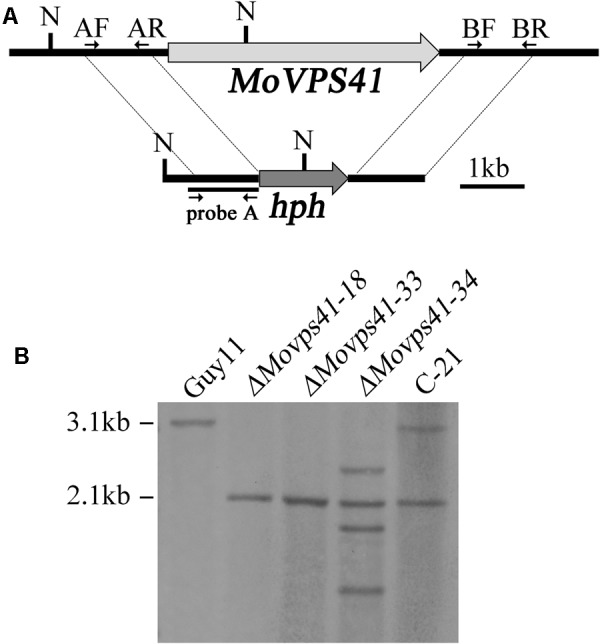
Generation of the *ΔMovps41* mutant. **(A)** The split-marker approach was used to replace the entire *MoVPS41* gene with the hygromycin resistance cassette (*hph*). Probe A is a PCR product amplified with primers AF (*VPS41*-AF) and AR (*VPS41*-AR). N: *Nco*I. **(B)** Southern blotting analysis with *Nco*I-digested genomic DNA of wild-type strain (Guy11), *ΔMovps41* mutant strains (*ΔMovps41-18, ΔMovps41-33*, and *ΔMovps41-34*) and complemented transformant (C-21).

**FIGURE 2:**
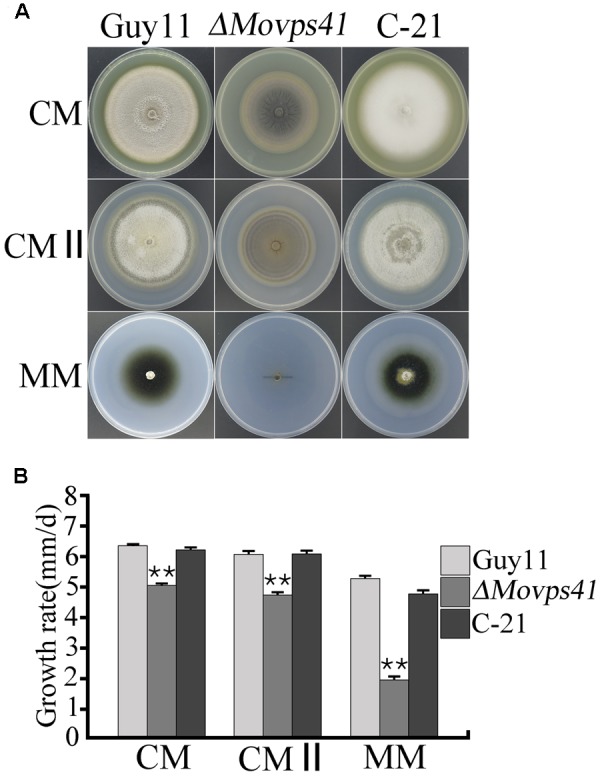
*MoVPS41* is required for vegetative growth in *M. oryzae*. **(A)** Shows growth characteristics of the *ΔMovps41*, wild-type and the C-21 complementation strain cultured for 10 days. **(B)** Portrays the significant difference in the vegetative growth of *ΔMovps41*, Guy11, and the C-21 strain on CM, CMII, and MM culture plates. Mean and standard error were calculated from three independent repeats. Asterisks represent significant difference between the Δ*Movps41* mutant and Guy11 with (*P* < 0.01).

Upon successful generation and confirmation of MoVPS41 deletion mutant (*ΔMovps4*-18, *ΔMovps4*-33, and *ΔMovps4*-34) mutant and complementation (**Figure 1**), we proceeded further to evaluate growth characteristics of the *ΔMovps41* mutant alongside the wild-type and the complemented strain on three different culture medium which includes; CM, CMII, and MM medium. Corresponding results obtained from this assay showed that the deletion of *MoVPS41* gene impacted negatively on the vegetative growth of *ΔMovps41* mutant and resulted in 20, 22, 64% reduction in the growth of the *ΔMovps41* mutant on CM, CMII, and MM medium, respectively (**Figures 2A,B**), additionally, we showed that the re-introduction of the wild-type *MoVPS41* gene into the *ΔMovps41* deletion mutant resulted in the generation of *ΔMovps41/MoVPS41* complemented strain (C-21) with growth characteristics similar to that of the wild-type strain (**Figure 2**). From these results we inferred that *M. oryzae MoVPS41* is deletable and further positioned that *MoVPS41* contribute significantly to efficient vegetative development of the rice blast fungus.

### Deletion of *MoVPS41* Blocks Sexual and Asexual Reproduction

**FIGURE 3:**
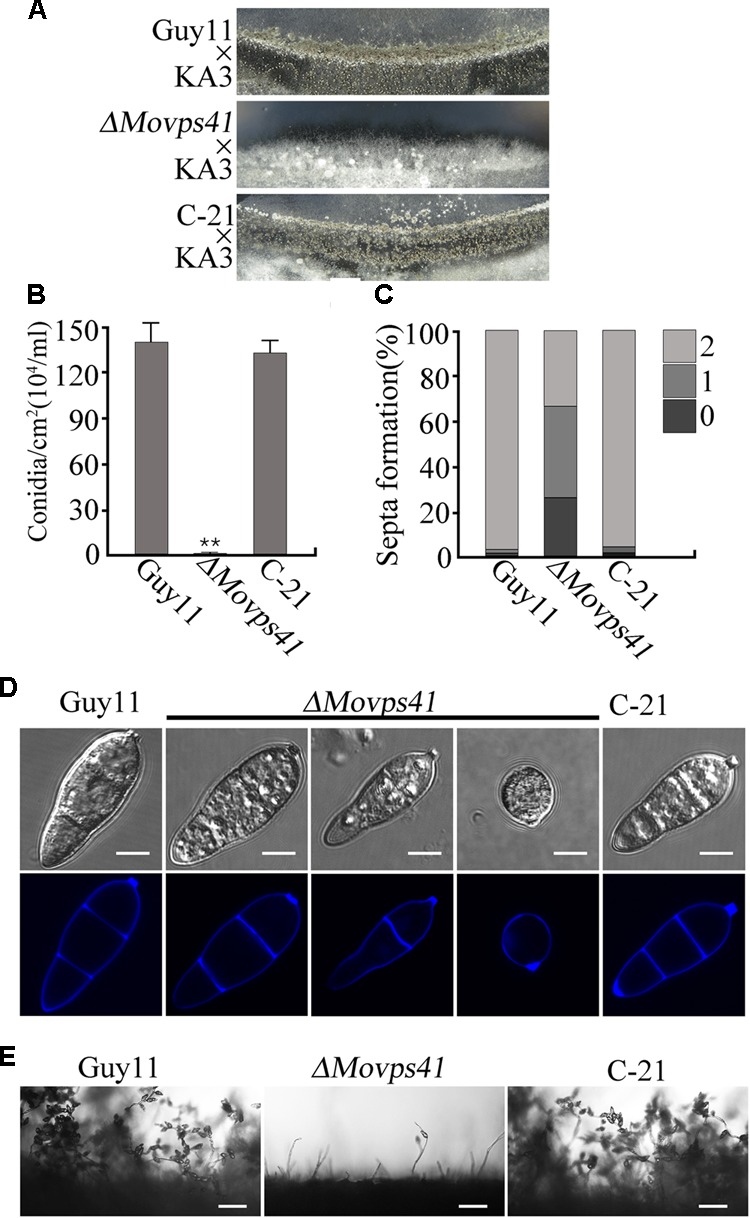
The *ΔMovps41* mutant showed severe defects in sexual and asexual reproductions. **(A)** In sexual reproduction experiments, no perithecia appeared at the cross of *ΔMovps41* × KA3, whereas Guy11 × KA3 and C-21 × KA3 formed numerous black perithecia at the junctions between fertile isolates on oatmeal medium. **(B)** Represent statistical record showing the number of conidia obtained from the *ΔMovps41* mutant, Guy11 and C-21 strain and, counted under microscope. The data was analyzed by ordinary one – ANOVA, error bars represent the standard deviation and double asterisks represent significant differences (*P* < 0.001). **(C)** Depicts quantification analysis of septa formation in the ΔMovps41 compared to the wild-type and C-21. **(D)** is a visual presentation of the various septation abnormalities associated with the *ΔMovps41* mutant relative to the wild-type Guy11 and C-21 Conidia were harvested and conidia septation assay was conducted by staining with CFW. Scale bar = 5 μm. **(E)** Displayed the disparities associated with the development of conidiophore between the *ΔMovps41* mutant, Guy11 and C-21 stains as observed under light microscope Scale bar = 50 μm.

In plant, Vps41 showed high expression activities in vegetative cells and sperm cells of pollen tubes during reproductive development and has been identified as a crucial factor regulating pollen tube–stigma interaction (Hao et al., 2016). To evaluate the possible contribution of MoVps41 to the sexual reproductive life of *M. oryzae*, we crossed wild-type strain Guy11 (MAT1-2) and mutant strain *ΔMovps41* and the *ΔMovps41/MoVPS41* complementation strain (C-21) with a standard mating type tester strain KA3 (MAT1-1) by culturing them on oatmeal (OMA) plates under optimum conditions required for perithecia production. Results obtained from this assay showed that Guy11 × KA3 and C-21 × KA3 crosses yielded numerous dark perithecia, whilst, no perithecia was observed under *ΔMovps41* × KA3 crosses (**Figure 3A**). We also examined asexual reproduction characteristics of the *MoVPS41* deletion mutant by assessing number and morphology of conidia produced by the *ΔMovps41* mutant. Corresponding results obtained from these conidiation assays showed that the quantity of conidia produced by *ΔMovps41* mutant was significantly low and constituted less than 0.1% of the quantity of conidia produced by the wild-type and C-21 complementation strain (**Figure 3B**). Additionally, most of the conidia produced by the *ΔMovps41* mutant exhibited septation defects and our records showed 40% of conidia produced by the *ΔMovps41* mutant are associated with single septum while conidium without septum accounts for 25.5% and the remaining 34.5% exhibited normal septation (**Figures 3C,D**). We also performed comparative conidiophore development assay to ascertain if the observed drastic reduction in the quantity of conidia produced by the *ΔMovps41* mutant has any relationship with conidiophore-genesis. Results, obtained from this assay showed that the deletion of *MoVPS41* equally triggered drastic reduction in the formation of conidiophore the *ΔMovps41* mutant (**Figure 3E**). From these result we posited that *MoVPS41* play crucial role in regulating conidiophore-genesis, conidia morphogenesis, sexual and asexual reproduction in *M. oryzae.*

### MoVps41 Is Required for Pathogenicity

**FIGURE 4:**
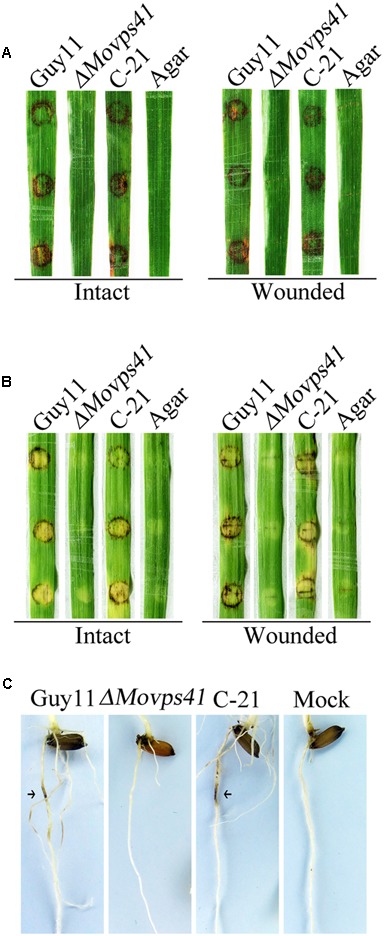
*MoVPS41* deletion rendered *ΔMovps41* non-pathogenic on rice and barley. **(A)** Intact (left panel) and wounded (right panel) rice leaves were inoculated with culture blocks of *ΔMovps41* mutant, wild-type and C-21. Inoculation with CMII medium blocks (Agar) was used as the negative control. Typical leaves were photographed 7 days after inoculation. **(B)** Intact (left panel) and wounded (right panel) barley leaves were inoculated with culture blocks of *ΔMovps41* mutant, wild-type and C-21. Inoculated leaves were photographed at 5-days post inoculation (5-dpi). **(C)** Root infection assay. Arrows indicate necrotic lesions. Pictures were taken 7 days after inoculation.

Because of deficiency in conidiation of the deletion mutants, we conducted pathogenicity assay by inoculating rice and barley seedlings with mycelium plugs obtained from the *ΔMovps41* mutant and the Guy11. This investigation showed that unlike the wild-type strain, the *ΔMovps41* mutant failed to induce symptoms of blast disease or lesions on susceptible rice seedling (CO39) host plant, while severe symptoms appeared 5-hpi on leaf tissues inoculated with culture block of wild-type and the C-21 complementation strain (**Figure 4A**). Because barley is also susceptible to *M. oryzae* blast infection, we decided to examine the virulence of *ΔMovps41* on barley leaves with or without wound. From this investigation we noticed that the *ΔMovps41* mutant consistently failed to induce blast lesion on both intact and injured barley leaves (**Figure 4B**). We additionally carried-out root infection assay with rice seedlings, and consistent with results obtained from leaf infection trials, the *ΔMovps41* mutant failed to trigger discoloration near the inoculation sites on rice root (**Figure 4C**). However, the wild-type and complemented C-21 strain readily induced extensive discoloration on rice root with under the same inoculation and incubation conditions, (**Figure 4C**), from these results we inferred that MoVPS41 mediated vacuolar fusion activities exerts great influence on the pathogenesis of the rice blast fungus by either directly or indirectly facilitating the secretion of virulence factors in *M. oryzae*.

### MoVps41 Is Important for Host Penetration Mediated by Hyphal Tip Appressorium-Like Structures

**FIGURE 5:**
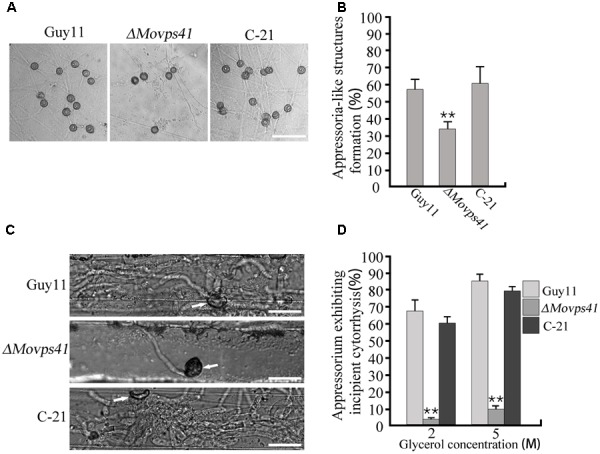
The *ΔMovps41* mutant produced dysfunctional hyphal tip appressorium-like structures at hyphal tips. **(A)** Appressorium-like structures formed on hydrophobic film by hyphal tips of wild-type (Guy11), *ΔMovps41* mutant (*ΔMovps41-18*) and complemented strain (C-21). Scale bar = 50 μm. **(B)** Bar graph representing quantification of the number of hyphae producing appressoria-like structures in the *ΔMovps41* mutant, wild-type and C-21. Asterisks represent significant difference between Guy11 and Δ*Movps41* mutant (*P* < 0.01). **(C)** Barley leaves inoculated with culture blocks of the *ΔMovps41* mutant, wild-type and C-21 were examined for the formation of invasive hyphae at 72 hpi. Arrows indicate appressorium-like structures. Scale bar = 20 μm. **(D)** Quantification of collapsed appressorium-like structures in the *ΔMovps41* mutant, wild-type Guy11 and C-21 strain. 2M and 5M glycerol were used for testing. Asterisks represent significant difference between Guy11 and Δ*Movps41* mutant (*P* < 0.01).

Records have shown that besides conidia mediated infection, *M. oryzae* also invades host tissues by deploying appressorium-like structures that develop at hyphal tips (Kong et al., 2013). However, from our pathogenicity assay we noticed that *ΔMovps41* failed to initiate hyphae mediated infection on intact leaves of barley and rice seedlings. In view of this observation, we decided to monitor the development of hyphae tip appressorium-like structures in the *ΔMovps41* mutant strain and the wild-type strain during growth on glass slide. Results obtained from this examination showed that the *ΔMovps41* mutant has no defect with regards to the formation of appressorium-like structures at hyphae (**Figures 5A,B**). We proceeded further to monitor the penetration abilities of the appressorium-like structures produced by *ΔMovps41* mutant by inoculating barley leaves with vegetative hyphae produced by the *MoVPS41* deletion mutant. Findings from microscopy examination after 48 hours post inoculation (hpi) revealed that the appressorium-like structures produced by the *ΔMovps41* mutant were unable to penetrate barley epidermal cells, whilst appressorium-like structures produced by the wild-type and complemented C-21 strains successfully penetrated barley epidermal cells and formed massive bulbous invasive hyphae inside the invaded epidermal cells (**Figure 5C**). Additional results obtained from incipient cytorrhysis assays surprisingly showed that the turgor generated by the appressorium-like structures produced by the *ΔMovps41* mutant was significantly higher than the level of turgor exhibited by the wild-type and complemented C-21 strains (**Figure 5D**). From these observations, we subsequently concluded that MoVps41 is required for enforcing the functionality of hyphae tip appressorium-like structures as a potent propagule for initiating hyphae mediated development of rice blast disease.

### MoVps41 Is Not Required for Appressorium Formation

**FIGURE 6:**
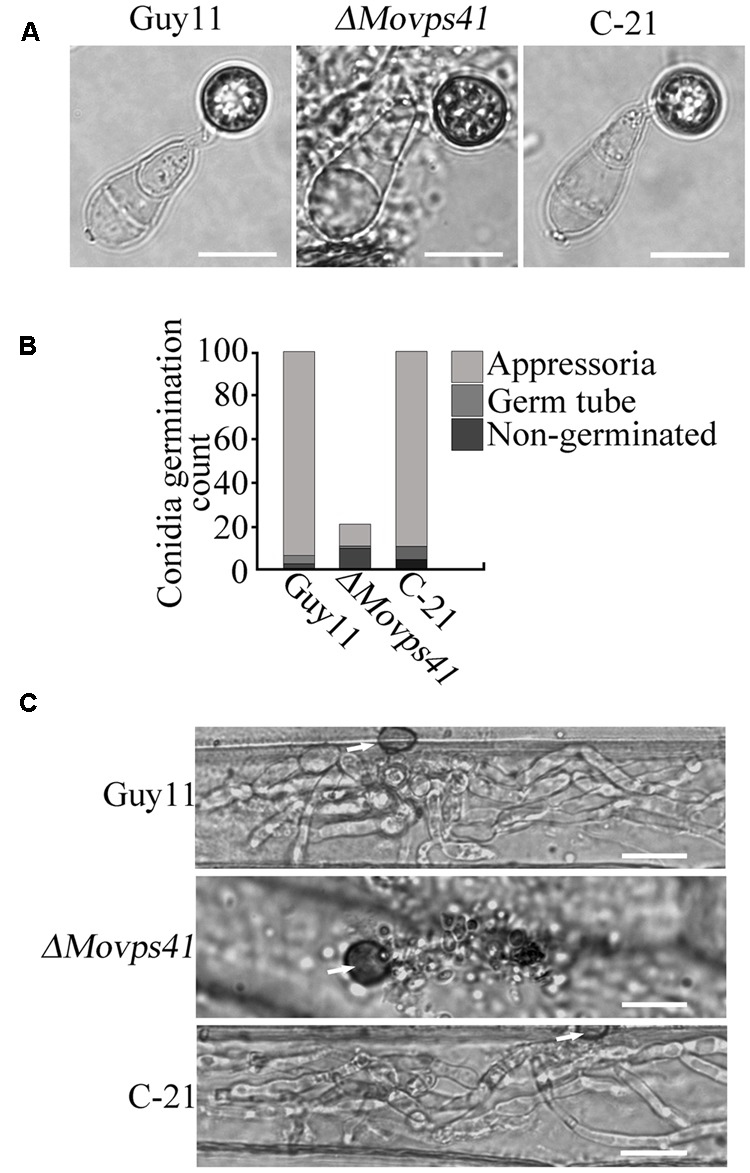
*MoVPS41* is dispensable of appressorium formation but indispensable of host penetration. **(A)** The conidia produced by the *ΔMovps41* deletion mutant is able to generate the appressorium. Scale bar = 2 μm. **(B)** The formation rate of appressorium of the *ΔMovps41* deletion mutant. The result is from three independent biological repeats. **(C)** Infection assay of conidia from *ΔMovps41* deletion mutant on barley epidermis cells. Scale bar = 20 μm.

To further establish the influence of *MoVPS41* on the development of conidia base appressorium, we accordingly carried-out appressorium formation assay on appressorium inducing hydrophobic surface with the few conidia obtained from the *ΔMovps41* mutant along the wild-type and the C-21 strain. This investigation showed that the *ΔMovps41* mutant could form intact appressoria of similar morphology with the appressorium produced by the wild-type and the C-21 strain (**Figures 6A,B**). However, results obtained from conidia mediated penetration assay conducted by inoculating barley leaves with conidia suspensions prepared with conidia obtained from the *ΔMovps41* mutant, the wild-type and C-21 strain, also showed that although the *ΔMovps41* mutant could form appressorium on host leaf surface, it was, however, unable to penetrate barley epidermal cells even after extended period of 72-hpi (**Figure 6C**). These results showed that *MoVPS41* is dispensable of appressorium formation in *M. oryzae*, taken these results together we proposed that *MoVPS41* contribute to the development of blast infection by facilitating efficient penetration of host tissues. However, the detailed molecular mechanism underlining this observation is still obscure at this stage and highlights the need for more comprehensive studies on the HOPS complex in the rice blast fungus in the future.

### MoVps41 Is Essential in Vacuole Fusion

**FIGURE 7:**
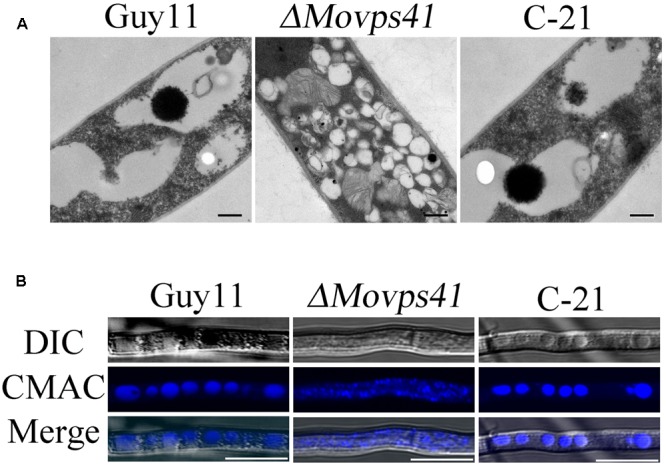
*MoVPS41* crucially regulate vacuolar morphogenesis and integrity in *M. oryzae*. **(A)** Numerous smaller vacuoles were present in hyphae of *ΔMovps41* mutant while large globular vacuoles were observed in wild-type and C-21complementation strain. Images were obtained with transmission electron microscope. Scale bar = 0.5 μm. **(B)** Vacuoles of hyphae were stained with CMAC. Numerous fragmented vacuoles were observed in the*ΔMovps41-18*, while large globular vacuoles were observed in Guy11 and C-21. Scale bar = 5 μm.

To ascertain the impact of *MoVPS41* deletion on vacuolar fusion process in the *ΔMovps41* mutant, we observed the morphological pattern of vacuoles in the vegetative hyphae of *ΔMovps41* mutant and the wild-type cultured in liquid CM media for 2-days with the aid of transmission electron microscopy (TEM). Results from our microscopy analysis identified numerous and characteristically smaller vacuoles in the *ΔMovps41* mutant. However, the vacuoles observed in the wild-type and the complemented strains were typically large (**Figure 7A**). To further confirm this observation, we stained hyphae produced by the *ΔMovps41*mutant, Guy11, and complemented strain with CMAC (7-amino-4-chloroethylcoumarin) dye which selectively stains fungal vacuoles. Our vacuole staining bio-assay also stained numerous smaller vacuoles in *MoVPS41* mutant while, few but larger size vacuoles were recorded for the wild-type, and the C-21 complemented strain (**Figure 7B**). These results adequately showed that *MoVPS41* is required for homotypic vacuole fusion processes in *M. oryzae*.

### MoVps41 Is Localized on the Vacuole Membrane

**FIGURE 8:**
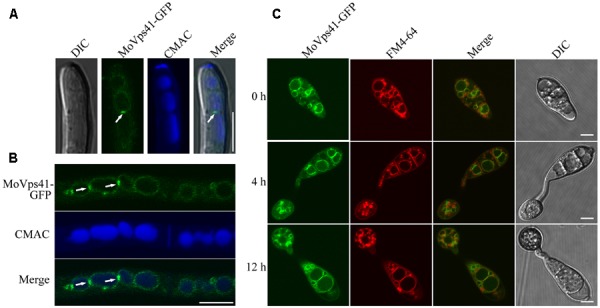
Subcellular localization of MoVps41 at different developmental stages of *M. oryzae*. **(A)** GFP signal cannot be observed in the apical region of hyphae but showed stronger GFP signals emerged at the interface between two vacuoles. CMAC dye was used to stain vacuole. Scale bar = 5 μm. **(B)** GFP fluorescent signal was observed in hypha of the *ΔMovps41*/*MoVPS41*-GFP strain cultured in liquid CM medium for 48 h with shaking at 150 rpm, 25°C. GFP signals were localized to vacuolar membranes and stronger GFP signals at the interface between two vacuoles. CMAC dye was used to stain vacuole. Scale bar = 10 μm. **(C)** Conidia from *ΔMovps41*/*MoVPS41*-GFP strain were incubated on the hydrophobic glass slides before examining fluorescent under microscope at defined time intervals of (0, 4, and 12 h). FM4-64 dye was used to stain vacuolar membranes. The merged panels show strong localization of MoVps41-GFP with vacuolar membranes. Scale bar = 5 μm.

To investigate the sub-cellular localization of MoVps41 protein, a MoVps41-GFP fusion construct generated in our study was transformed into the *ΔMovps41* mutant. After screening by PCR, one transformant MOVps41-GFP expressing the MoVps41-GFP construct under control of its native promoter was identified. Similar to the complemented strain C-21, all defects displayed in the *ΔMovps41* mutant were rescued in the *ΔMovps41*/MoVps41-GFP transformant MOVps41-GFP, indicating that the fusion constructs MoVps41-GFP is functional. We used CMAC to stain the hyphal vacuoles of *ΔMovps41*/MoVps41-GFP transformant MOVps41-GFP and observed the GFP signal under an epifluorescence microscope. MoVps41-GFP signals were observed on the membrane of the vacuoles, and the signals were stronger at the interface between two vacuoles (**Figures 8A,B**), suggesting that MoVps41 is involved in the homotypic fusion of vacuoles. We also investigated the localization of MoVps41 during appressorium formation on hydrophobic surface and showed that MoVps41 retains its localization to vacuolar membrane during conidia germination and appressoria formation. Furthermore, we examined the localization pattern of MoVps41 during progressive endocytosis and endosomal trafficking processes in conidia, germ tubes and appressoria at time points (0, 4, and 12 h) of appressorium development on a hydrophobic surface by staining with endocytosis oriented dye FM4-64 which stains depict the transport activities occurring on vacuolar membrane during progressive active endocytosis. Consistent with the localization observed for hyphae, conidia, germ tube and appressoria, this also showed FM4-64 epifluorescence signal co-localized with green Florence protein (GFP) signal on vacuolar membranes and exhibited the strongest expression signal at membrane contact sites between two vacuoles (**Figure 8C**). These results all suggest that MoVps41 is involved in homotypic vacuolar fusion.

### *MoVPS41* Deletion Mutants Are Highly Sensitive to Metal Ions

**FIGURE 9:**
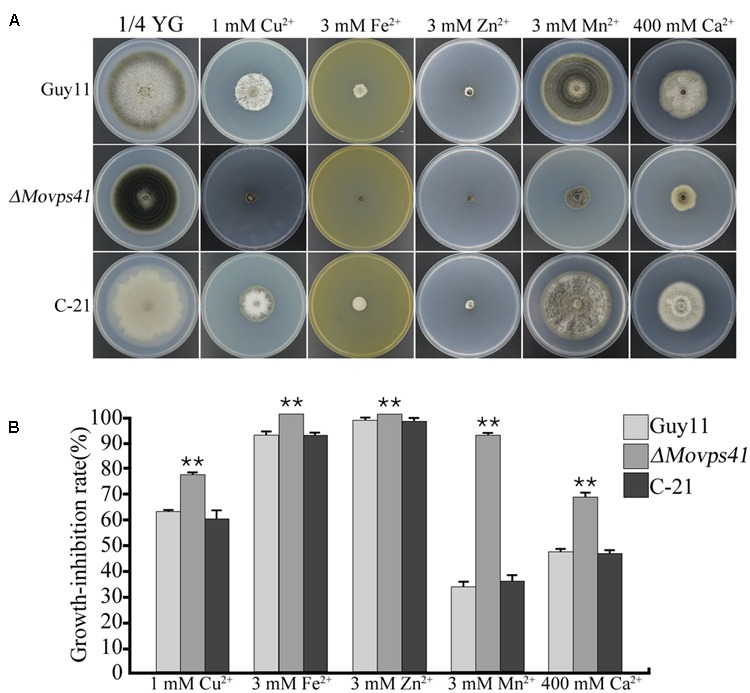
*ΔMovps41* mutant exhibited high metal ion sensitivity. **(A)** The colony morphology of wild-type (Guy11), *ΔMovps41* mutant (*ΔMovps41-18*) and complemented transformant (C-21) on 1/4 YG plates supplemented with 1 mM Cu^2+^, 3 mM Zn^2+^, 3 mM Mn^2+^, 3 mM Fe^2+^, and 400 mM Ca^2+^ at 25°C for 10 days. **(B)** The growth inhibition rate of wild-type (Guy11), *ΔMovps41* mutant (*ΔMovps41-18*) and complemented transformant (C-21) on 1/4 YG plates supplemented with 1 mM Cu^2+^, 3 mM Zn^2+^, 3 mM Mn^2+^, 3 mM Fe^2+^, and 400 mM Ca^2+^ at 25°C for 10 days. The experiment was performed at least three biological repeats. Asterisks represent significant difference between Guy11 and Δ*Movps41* mutant (*P* < 0.01).

Generally, metal ions, including Ca^2+^, Cu^2+^, Fe^2+^, Mn^2+^, and Zn^2+^ play an essential physiological role in the development of fungi (Wang et al., 2003). However, research demonstrations have shown that, the intracellular accumulation of these cations beyond certain physiological limit turn to impact negatively (metal ion toxicity) on fungal growth and development. Sequestering activities of the vacuole play an important role in shielding cells from the harmful effects of toxic metal ions and also crucially mediate the detoxification of these metal ions (Ramsay and Gadd, 1997). To examine the contribution of MoVps41 in vacuolar sequestering and subsequent detoxification of metal ions, we monitored the vegetative growth *ΔMovps41* mutant, Guy11 wild-type and the C-21 complemented strain on 1/4 YG growth medium supplemented with defined concentration of divalent metal ions. From this investigation, we observed that *ΔMovps41* mutant was highly sensitive to all the metal ions tested and accounted for about 68 to 92% growth inhibition in the *ΔMovps41* mutant with 1 mM Cu^2+^ exerting greatest biological growth inhibitory effect (71.2%) on *ΔMovps41* mutant compared to 51 and 54% growth inhibition recorded for C-21 complemented strain and Guy11, respectively (**Figures 9A,B**). These results imply that MoVps41 plays a crucial role in ensuring cellular metal ions homeostasis by facilitating vacuolar mediated sequestering and detoxification of metal ions.

### *ΔMovps41* Deletion Mutant Is Hypersensitive to Vesicular Transport Inhibitors, Osmotic, Oxidative, and Cell Wall Stresses

**FIGURE 10:**
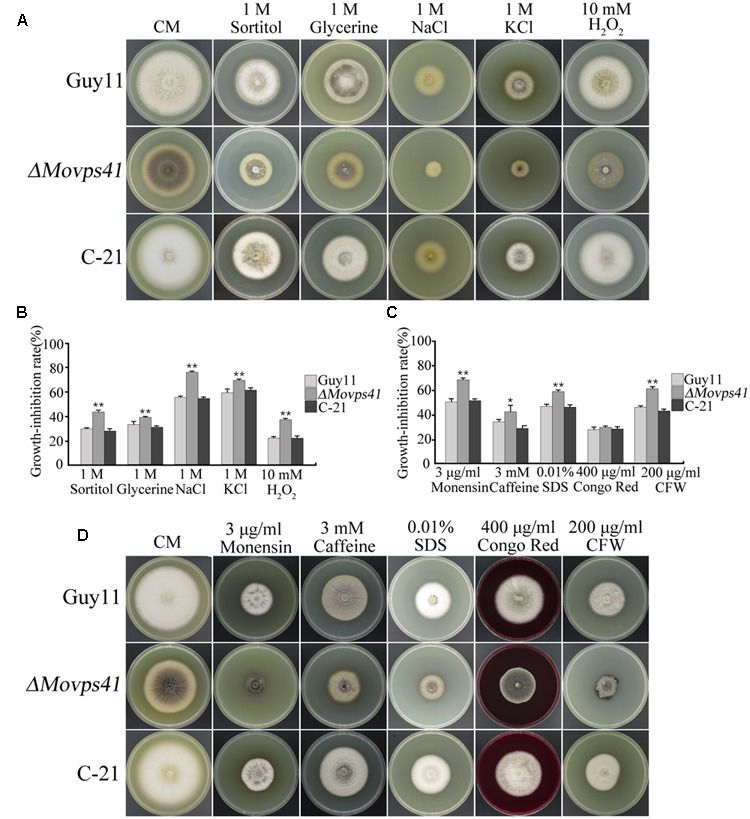
*ΔMovps41* mutant displayed high sensitivity toward osmotic and oxidative stress inducing osmolytes. **(A)** Portrays vegetative growth attributes of *ΔMovps41* mutant, wild-type and C-21 complementation strain on CM plates supplemented with (1 M sorbitol, 1 M glycerine, 1 M NaCl, and 1 M KCl), **(B)** Shows the statistical analysis of *ΔMovps41* mutant, wild-type and C-21 complementation strain growing on CM plates supplemented with (1 M sorbitol, 1 M glycerine, 1 M NaCl, and 1 M KCl), The experiment was performed at least three biological repeats. Error bar represent the standard deviation between the *ΔMovps41* mutant, wild-type and C-21 complementation strain and double asterisks represent *P* < 0.01. **(C)** Statistical computation of growth response of *1Movps41* mutant, wild-type and C-21 complementation strain on CM plates supplemented with osmolytes (0.01% SDS, 400 μg/mL Congo red, and 200 μg/mL Calcofluor white (CFW). The experiment was performed at least three biological repeats. Single asterisks represent *P* < 0.05, whilst double asterisks represent (*P* < 0.01). **(D)** Displays the growth performance of *ΔMovps41* mutant, wild-type and C-21 complementation strain on CM plates supplemented with cell wall stress inducing osmolytes (0.01% SDS, 400 μg/mL Congo red, and 200 μg/mL Calcofluor white (CFW).

The prevailing osmotic gradient across organelles and membranes has been cited as crucial factors modulating the rate of membrane fission and fusion processes (Brett and Merz, 2008). Accumulating evidence from previous research findings has shown that the signaling activities of HOPS complex proteins are enhanced in response to oxidative stress induced by different types of osmolytes (Brett and Merz, 2008). We therefore evaluated the sensitivity of *ΔMovps41* mutant to both osmotic and oxidative stresses by culturing the *ΔMovps41* mutant, Guy11 and the C-21 strains on CM plates supplemented with various types of osmotic and oxidative stress inducing osmolytes including; 1 M glycerine, 1 M sorbitol, 1 M NaCl, 1 M KCl, and 10 mM H_2_O_2_. This investigation revealed that *ΔMovps41* mutant display high sensitivity toward all the osmolytes tested, however, we noticed that NaCl, KCl, and CFW exerted the greatest inhibitory effect on the *ΔMovps41* mutant (**Figures 10A,B**). Additionally, we investigated the role of *MoVPS41* in enforcing cell wall integrity by monitoring the comparative growth performance of the *ΔMovps41* mutant, Guy11 and the C-21 strains on CM plates supplemented with 400 μg/mL Congo red, 0.01% SDS, Compared to the wild-type Guy11 and the C-21 complemented strain, the *ΔMovps41* mutant was moderately sensitive to SDS (**Figures 10C,D**). However, there was no significant difference observed in the growth of *ΔMovps41* mutant, Guy11 and the C-21 complemented strain on CM plates supplemented with 400 μg/mL Congo red (**Figure 10C**). These results demonstrate that MoVps41 plays an essential role in stabilizing membrane integrity in the rice blast fungus under oxidative stress conditions.

## Discussion

The homotypic fusion and vacuole protein sorting (HOPS) complex, which is made up of VPS-C complex (Vps11, Vps16, Vps18, and Vps33) and two accessory subunits Vps39 and Vps41 are on record as the first tether complex identified in the endocytic system (Runkle, 2012; Ellis, 2014). Insight gained from previous studies showed that the HOPS complex constitute a crucial cellular machinery that promotes the homotypic fusion of vacuoles (Krämer and Ungermann, 2011), it has been established that Vam2∖Vps41, one of the two accessory subunits constituting the HOPS complex functions as an effector that binds to Ypt7∖Rab7 and mediates vacuolar related fusion activities, including; homotypic vacuolar fusion, late endosome vacuolar fusion and auto-phagosome vacuolar fusion in yeast (Kleine Balderhaar et al., 2013). Although it has been shown that Vps41 coupled homotypic fusion enhances growth, stress tolerance, and virulence of many pathogenic microbes (Veses et al., 2009; Möst, 2014; Smith, 2015), the influence of MoVps14 on general growth and pathogenesis of the rice blast fungus has not been explored.

In this study, we identified *M. oryzae* specific Vps41 gene (MoVps41) and used functional genetic tools to elucidate the physiological and pathological importance of MoVps41 in the rice blast fungus. Assessment of vegetative growth characteristics of *ΔMovps41* mutant generated in this study showed that MoVPS41 deletion caused significant reduction in the vegetative growth of the *ΔMovps41* mutant on both nutrient deficient and nutrient sufficient growth medium. This is in conformity with earlier research findings, which showed that the deletion of Vps41 in yeast and Cryptococcus impacted negatively on the vegetative growth of the respective deletion mutant (Kim and Klionsky, 2000; Hu et al., 2015). These observations coupled with the knowledge that Vps41 functions as an effector protein that binds to Ypt7∖Rab7 which in turn interacts with major vesicle biogenesis machinery Yck3∖AP-3 complex (Sato et al., 2000), subsequently informed our conclusion that MoVps41 contributes to optimum vegetative development of *M. oryzae* by promoting vacuolar sorting processes required for efficient biogenesis.

Furthermore, our investigations revealed that the deletion of MoVps41 disrupted the progression of sexual reproduction and rendered the *ΔMovps41* mutant unable to produce perithecia even in the presence of fertile opposite mating type strain. We also showed that MoVps41 deletion did not only abolish sexual reproduction in the defective mutants but also drastically reduced the number of conidia produced by the *ΔMovps41* mutant, from these results we deduced that MoVps41 plays an indispensable role in the sexual and asexual reproductive life of *M. oryzae* by modulating the ability of the vacuoles to regulate nutrient availability as well as trans-membrane trafficking of vesicles and cargoes required for enhancing the fertility and reproduction in the rice blast fungus. This position is firmly confirmed by previous plant science research studies which showed that in flowering plants Vps41 essentially promotes sexual reproduction by facilitating the fertilization, germination and as well mediates the transfer of mature pollens for efficient pollen tube–stigma interaction and exhibited high expression pattern in actively dividing vegetative cells (Hao et al., 2016). Putting together, our investigation showed for the first time that MoVps41 like its plants counterparts play an essential role in regulating the initiation and progression of sexual and asexual reproduction in the rice blast fungus.

Many pathogenic microbes invade and successfully colonize host cells by secreting non-classically secreted proteins as effectors to suppress or evade host defense (Thomma et al., 2011). Research evidence available have shown that the successful secretion of non-secretion peptide effector proteins as virulence factors by pathogenic microorganisms, including plant pathogenic fugal species is mediated by the vacuolar complex through homotypic fusion and endocytic transport activities of vacuoles (Wong et al., 2013; Teh and Hofius, 2014; Escoll et al., 2016). MoVps41 is a component of the HOPS complex and play crucial functions in promoting homotypic vacuolar fusion and biogenesis (Nickerson et al., 2009), our study revealed that targeted gene replacement of *M. oryzae* VPS41 impacted negatively on the pathogenesis of the rice blast fungus and rendered the Δ*Movps41* mutant non-pathogenic on non-host and susceptible host plant, this observation in conjunction with previous findings which showed that the inactivation of Vps41 in *Candida albicans* and *Cryptococcus neoformans* equally impaired the pathogenicity and virulence of the respective defective mutants (Liu et al., 2006), forms the basis for our conclusion that MoVps41 contribute positively to the development of blast disease by promoting vacuolar mediated secretion of non-classical effector proteins during host-pathogen interaction and confirms previous proposal suggesting that the fusion activities of the HOPS complex crucially supports the progression of basic but vital developmental processes such as proper colony growth, efficient generation of aerial hyphae and sporulation (Ramanujam et al., 2013).

Current knowledge with regards to underlining mechanisms facilitates efficient differentiation of appressorium into penetration-peg to promote successful rupturing and invasion of host cells by the rice blast fungus has shown that the accumulation of glycerol in the appressoria cell constitute a pre-requisite for generating enormous turgor required to promote the development of penetration-peg to initiate host penetration process (Dagdas et al., 2012; Dagdas, 2013; Zheng H. et al., 2015; Zheng W. et al., 2015). However, we observed that hyphae tip appressorium-like structures produced by Δ*Movps41* mutant accumulated higher turgor pressure than the wild-type and C-21 but failed to penetrate barley epidermal cells. Although, this result sharply contradicts our expectation, we however, proposed that MoVps41 may influence the influence host penetration process by turgor pressure regulating influx balance between water and glycerol. More so, since the development of penetration-peg occurs as a function of negative membrane curvature which requires additional energy barriers (Bourett and Howard, 1990; Egan et al., 2007; Stachowiak et al., 2013), we subsequently deduced that the disruption of MoVps41 disrupted the pressure dynamics required for development of penetration-pegs by triggering steady accumulation of appressorium glycerol level.

Homotypic vacuole to vacuole fusion represents an efficient biosynthetic cellular transport system to the vacuole (LaGrassa and Ungermann, 2005), Vps41 belongs to the HOPS complex group of proteins that are known to play a vital role in promoting tethering, docking and more importantly homotypic vacuolar fusion as well as *trans*-membrane trafficking processes in a wide range of organisms (Krämer and Ungermann, 2011). In yeast, Vps41 is required for successful vacuolar fusion, maintaining normal vacuolar morphology and integrity, subsequently, the deletion of Vps41 (*Δvps41)* in yeast resulted in the formation of highly fragmented vacuole (Radisky et al., 1997; Auffarth et al., 2014). Similarly Vacuole staining and microscopy examinations conducted with *ΔMovps41* mutant generated in this study also revealed high incidence of vacuole fragmentation. In line with these observations, we reasoned that Vps41 mediated stabilization of vacuolar morphology and vacuolar fusion is an evolutionarily conserved function across kingdoms.

Finally, our study further revealed that *ΔMovps41* mutant are highly sensitive to most osmotic stress or oxidative stress inducing osmolytes including; glycerine, sorbitol, NaCl, KCl and H_2_O_2_ and subsequently resulted in significant reduction in the vegetative growth of *ΔMovps41* on media supplemented with glycerine, sorbitol, NaCl, KCl and H_2_O_2_. Additionally, we noticed that the *ΔMovps41* mutant displayed much higher sensitivity toward caffeine and monensin administered in growth media as vesicular transport inhibitors (Kuismanen et al., 1992). Our analysis further showed that the *ΔMovps41* mutant have almost an intact cell wall integrity, and was less sensitive to cell wall stress inducing osmolytes these results are in tandem with previous research demonstrations indicating that in yeast MoVps41 activities increased in response to oxidative stress (Chang and Neufeld, 2010). In line with these results, we arrived at the conclusion that MoVps41 contributes to enforcing cell membrane integrity and intracellular vesicle transport by enhancing efficient and timely vacuolar sequestering of toxic metabolites in *M. oryzae*.

## Materials and Methods

### Fungal Strains and Culture Conditions

The wild-type strain Guy11 and all transformants generated in this study were cultured on complete medium plates (CM: 0.6% yeast extract, 0.6% casein hydrolysate, 1% sucrose, 1.5% agar) at 25°C as described previously (Chen et al., 2008). Additional growth media used in this study, including minimal medium (MM: 6 g NaNO_3_, 0.52 g KCl, 0.52 g MgSO_4_, 1.52 g KH_2_PO_4_, 10 g glucose and 15 g agar in 1 L distilled water), 1/4 YG medium (1.3 g yeast extract, 5 g glucose, 15 g agar in 1 liter of distilled water) and oatmeal agar medium (OMA: 30 g oatmeal and 15 g agar in 1 L distilled water). For sexual reproduction, strains were cultured on oatmeal agar medium for 4 weeks (Karthikeyan and Gnanamanickam, 2008). For conidiation, the strains were cultured on rice bran medium (2% rice bran, 1.5% agar, pH 6.0) with a 12 h photophase for 7 days and removed all aerial hyphae and incubated the cultures under light for additional 48 h (Zhong et al., 2015). For DNA extractions and protoplast preparation, strains were grown in the liquid CM in a 150 rpm shaker at 25°C for 2–3 days. Sensitivity assay was conducted by culturing the respective strains on 1/4YG or CM plates supplemented with metal ionic stresses (1 mM Cu^2+^, 3 mM Zn^2+^, 3 mM Mn^2+^, 3 mM Fe^2+^ and 400 mM Ca^2+^), vesicular transport inhibitors (3 mM caffeine and 3 μg/mL monensin), osmotic stresses (1 M sorbitol, 1 M glycerine, 1 M NaCl, and 1 M KCl), oxidative stresses (10 mM H_2_O_2_), and cell wall stresses [0.01% SDS, 400 μg/mL Congo red and 200 μg/mL Calcofluor white (CFW)].

### Generation of the *ΔMovps41* Deletion Mutants and Complemented Transformants

Split-marker approach (Goswami, 2012; Zheng et al., 2012) was applied to delete the *MoVPS41* gene in *M. oryzae*. A 1.1-kb upstream and a 1.2-kb downstream flanking fragments of *MoVPS41* were amplified with primer pairs VPS41-AF/AR and VPS41-BF/BR (Supplementary Table S1), respectively. The resulting PCR products were ligated with hygromycin phosphotransferase (*hph*) cassette fragments amplified with primers HYG/F+HY/R and YG/R+HYG/R (Supplementary Table S1) by overlapping PCR. For fungal transformation, protoplast preparation and polyethylene glycol (PEG)-mediated transformation of *M. oryzae* was performed as described by Zhang et al. (2016). Hygromycin-resistant transformants were screened by PCR with primer pairs VPS41-OF+VPS41-OR and VPS41-UA+H853 (Supplementary Table S1). Subsequently, putative *ΔMovps41* mutants were analyzed by Southern blotting to confirm the gene replacement event. For complementation assays, a fragment containing the entire *MoVPS41* gene, a 2-kb upstream and 1.4-kb downstream sequences was amplified with primers VPS41-CF and VPS41-CR (Supplementary Table S1). The resulting PCR products were co-transformed into *ΔMovps41* mutant with pKNT vector. G418-resistant transformants *ΔMovps41/MoVPS41* were confirmed by PCR and Southern blotting analysis.

### DNA Manipulation and Southern Blotting Analysis

For DNA extraction, genomic DNA was extracted from wild-type strain Guy11, *ΔMovps41* deletion mutants and C-21 complemented transformants with the use of CTAB method described by Wang et al. (2017). For Southern blotting analysis, genomic DNA aliquots of 5 μg were completely digested with *Nco*I enzyme and separated with a 0.7% agarose gel and transferred onto Hybond N+ membrane (Amersham Pharmacia Biotech). The “A-fragments” were amplified with VPS41-AF and VPS41-AR primers (**Figure 1** and Supplementary Table S1) and labeled by digoxin (DIG) as probe A. For southern blot analysis, probe labeling, hybridization and detection were performed with DIG High Prime DNA labeling and Detection Starter Kit I (Roche Applied Science), according to the instruction manual.

### Phenotype Assay

Conidia harvested from rice bran agar were counted by using hemocytometer and photographed by Olympus BX51 microscope (Olympus Corporation, Japan). Three independent experiments were performed with three replicates each time. For fertility assays, the strains crossed with strain KA3 were cultured on OMA at 25°C for the 7 days with a 12-h photophase and then placed under continuous white light at 16°C for 4 weeks. The border between the mated individuals was examined for the capacity to form perithecia. This experiment was repeated three times. Both leaf and root inoculation was carried-out by inoculating intact and injured barley (Gold Promise) and rice (CO39) leaves with a block media carrying mycelia before incubating them in a moist chamber at 25°C for 5–7 days as described by (Yan et al., 2011).

### Assays for the Formation and Penetration of Appressorium-Like Structures

The development of appressorium-like structures at hyphal tips was assayed in accordance to the procedures described by Kong et al. (2013). To induce appressorium-like structure formation, culture blocks of 4-day-old CM cultures were placed on hydrophobic surfaces, and incubated in moist chamber for 72 h in dark. For penetration assays, the leaves of 10-day-old seedling of barley (Gold Promise) were inoculated with culture blocks of 4-day-old CM cultures for 48 h and subsequent penetration and *in vivo* development invasive hyphae was examined in line with procedures described by (Liu et al., 2010; Kong et al., 2013).

### Generation of the MoVps41-GFP Fusion Construct

To generate the *MoVPS41*-GFP fusion construct, a 2.2-kb DNA fragment containing the entire *MoVPS41* gene and its promoter region was amplified with primers VPS41-GF and VPS41-GR (Supplementary Table S1) and inserted into the *APa*I/*Hind*III site of pKNTG vector. The resulting fusion construct *MoVPS41*-GFP was transformed into *ΔMovps41* mutant through PEG-mediated transformation. G418-resistant transformants were screened by PCR with primers VPS41-ORF and VPS41-GR (Supplementary Table S1) and examined for GFP signals.

### Staining and Microscopy Assays

For transmission electron microscopy, observation was carried out as previously described (Wang et al., 2011). For vacuolar staining, hyphae were prepared by growing fragmented mycelia in liquid CM for 3-days at 25°C with shaking at 150 rpm and inoculated with a vacuolar luminal dye CMAC (7-amino-4-chloromethylcoumarin, Sigma–Aldrich) at a final concentration of 10 μM for 20 min as previously described (Ohneda et al., 2002; Menke, 2011). Germinating conidia (0h, 4h and 12 h) on hydrophobic surface were incubated with 32 μM FM4-64 for 30 min to stain the vacuolar membrane. The vacuolar morphology of vegetative hyphae and germinating conidia were observed under the Nikon TiE system (Nikon, Japan).

## Author Contributions

The experiments was conceived and designed: XZ, GW, and ZW. The experiments Performed by: XZ, GW, CY, JH, and XC. Wrote the paper: XZ, GW, JZ, GL, JN, and ZW.

## Conflict of Interest Statement

The authors declare that the research was conducted in the absence of any commercial or financial relationships that could be construed as a potential conflict of interest.

## Footnotes

1 https://www.broadinstitute.org/scientific-community/science/projects/fungal-genome-initiative/magnaporthe-comparative-genomics-proj

## References

[B1] Ariosa A. R. Klionsky D. J. (2016). Autophagy core machinery: overcoming spatial barriers in neurons. *J. Mol. Med.* 94 1217–1227. 10.1007/s00109-016-1461-9 27544281 PMC5071157

[B2] Auffarth K. Arlt H. Lachmann J. Cabrera M. Ungermann C. (2014). Tracking of the dynamic localization of the Rab-specific HOPS subunits reveal their distinct interaction with Ypt7 and vacuoles. *Cell. Log.* 4:e29191 10.4161/cl.29191 PMC4156483 25210650

[B3] Barlowe C. K. Miller E. A. (2013). Secretory protein biogenesis and traffic in the early secretory pathway. *Genetics* 193 383–410. 10.1534/genetics.112.142810 23396477 PMC3567731

[B4] Bourett T. M. Howard R. J. (1990). In vitro development of penetration structures in the rice blast fungus *Magnaporthe grisea*. *Can. J. Bot.* 68 329–342. 10.1139/b90-044

[B5] Brett C. L. Merz A. J. (2008). Osmotic regulation of Rab-mediated organelle docking. *Curr. Biol.* 18 1072–1077. 10.1016/j.cub.2008.06.050 18619842 PMC2807628

[B6] Chang Y.-Y. Neufeld T. P. (2010). Autophagy takes flight in *Drosophila*. *FEBS Lett.* 584 1342–1349. 10.1016/j.febslet.2010.01.006 20079355 PMC2843783

[B7] Chaudhari P. Ahmed B. Joly D. L. Germain H. (2014). Effector biology during biotrophic invasion of plant cells. *Virulence* 5 703–709. 10.4161/viru.29652 25513771 PMC4189876

[B8] Chen C. H. (2016). *Characterization of VPS33B and VPS16B in Megakaryocyte and Platelet I-granule Biogenesis*. Masters, University of Toronto Toronto, ON.

[B9] Chen J. Zheng W. Zheng S. Zhang D. Sang W. Chen X. (2008). Rac1 is required for pathogenicity and Chm1-dependent conidiogenesis in rice fungal pathogen *Magnaporthe grisea*. *PLoS Pathog.* 4:e1000202 10.1371/journal.ppat.1000202 PMC2575402 19008945

[B10] Dagdas Y. F. Yoshino K. Dagdas G. Ryder L. S. Bielska E. Steinberg G. (2012). Septin-mediated plant cell invasion by the rice blast fungus. *Magnaporthe oryzae*. *Science* 336 1590–1595. 10.1126/science.1222934 22723425

[B11] Dagdas Y. F. (2013). *The Role of Cellular Morphogenesis in the Pathogenicity of the Rice Blast Fungus Magnaporthe oryzae.* Doctor of Philosophy, University of Exeter Exeter.

[B12] Dean R. Van Kan J. A. Pretorius Z. A. Hammond-Kosack K. E. Di Pietro A. Spanu P. D. (2012). The Top 10 fungal pathogens in molecular plant pathology. *Mol. Plant Pathol.* 13 414–430. 10.1111/j.1364-3703.2011.00783.x 22471698 PMC6638784

[B13] Dou D. Zhou J.-M. (2012). Phytopathogen effectors subverting host immunity: different foes, similar battleground. *Cell Host Microbe* 12 484–495. 10.1016/j.chom.2012.09.003 23084917

[B14] Egan M. J. Wang Z.-Y. Jones M. A. Smirnoff N. Talbot N. J. (2007). Generation of reactive oxygen species by fungal NADPH oxidases is required for rice blast disease. *Proc. Natl. Acad. Sci. U.S.A*. 104 11772–11777. 10.1073/pnas.0700574104 17600089 PMC1913907

[B15] Ellis K. L. (2014). *Molecular Mechanisms of Notochord Vacuole Formation and Their Role in Zebrafish Development.* Durham, NC: Duke University.

[B16] Escoll P. Mondino S. Rolando M. Buchrieser C. (2016). Targeting of host organelles by pathogenic bacteria: a sophisticated subversion strategy. *Nat. Rev. Microbiol.* 14 5–19. 10.1038/nrmicro.2015.1 26594043

[B17] Goswami R. S. (2012). Targeted gene replacement in fungi using a split-marker approach. *Plant Fungal Pathog. Methods Protoc.* 835 255–269. 10.1007/978-1-61779-501-5_16 22183659

[B18] Guerra F. Bucci C. (2016). Multiple roles of the small GTPase Rab7. *Cells* 5:34 10.3390/cells5030034 PMC5040976 27548222

[B19] Hammer J. A. Sellers J. R. (2012). Walking to work: roles for class V myosins as cargo transporters. *Nat. Rev. Mol. Cell Biol.* 13 13–26. 10.1038/nrm3248 22146746

[B20] Hao L. Liu J. Zhong S. Gu H. Qu L.-J. (2016). AtVPS41-mediated endocytic pathway is essential for pollen tube–stigma interaction in *Arabidopsis*. *Proc. Natl. Acad. Sci. U.S.A*. 113 6307–6312. 10.1073/pnas.1602757113 27185920 PMC4896696

[B21] Hong W. Lev S. (2014). Tethering the assembly of SNARE complexes. *Trends Cell Biol.* 24 35–43. 10.1016/j.tcb.2013.09.006 24119662

[B22] Hu G. Mcquiston T. Bernard A. Park Y.-D. Qiu J. Vural A. (2015). A conserved mechanism of TOR-dependent RCK-mediated mRNA degradation regulates autophagy. *Nat. Cell Biol.* 17 930–942. 10.1038/ncb3189 26098573 PMC4528364

[B23] Janková Drdová E. (2017). *The Secretory Vesicles Tethering Complex Exocyst and the Auxin Transport Polarization.* Ph.D. thesis, Charles University Prague.

[B24] Kankanala P. Czymmek K. Valent B. (2007). Roles for rice membrane dynamics and plasmodesmata during biotrophic invasion by the blast fungus. *Plant Cell* 19 706–724. 10.1105/tpc.106.046300 17322409 PMC1867340

[B25] Karthikeyan V. Gnanamanickam S. (2008). Determining the fertility status of setaria infecting *Magnaporthe grisea* isolates with standard testers and identification of tolerant cultivar of *Setaria italica*. *Mycopathologia* 166 227–233. 10.1007/s11046-008-9141-0 18597182

[B26] Kim J. Klionsky D. J. (2000). Autophagy, cytoplasm-to-vacuole targeting pathway, and pexophagy in yeast and mammalian cells. *Ann. Rev. Biochem.* 69 303–342. 10.1146/annurev.biochem.69.1.303 10966461

[B27] Kleine Balderhaar H. J. Lachmann J. Yavavli E. Bröcker C. Lürick A. Ungermann C. (2013). The CORVET complex promotes tethering and fusion of Rab5/Vps21-positive membranes. *Proc. Natl. Acad. Sci. U.S.A*. 110 3823–3828. 10.1073/pnas.1221785110 23417307 PMC3593874

[B28] Klionsky D. Herman P. K. Emr S. (1990). The fungal vacuole: composition, function, and biogenesis. *Microbiol. Rev.* 54 266–292. 2215422 10.1128/mr.54.3.266-292.1990 PMC372777

[B29] Kong L. A. Li G. T. Liu Y. Liu M. G. Zhang S. J. Yang J. (2013). Differences between appressoria formed by germ tubes and appressorium-like structures developed by hyphal tips in *Magnaporthe oryzae*. *Fungal Genet. Biol.* 56 33–41. 10.1016/j.fgb.2013.03.006 23591122

[B30] Krämer L. Ungermann C. (2011). HOPS drives vacuole fusion by binding the vacuolar SNARE complex and the Vam7 PX domain via two distinct sites. *Mol. Biol. Cell* 22 2601–2611. 10.1091/mbc.E11-02-0104 21613544 PMC3135484

[B31] Kuismanen E. Jantti J. Makiranta V. Sariola M. (1992). Effect of caffeine on intracellular transport of Semliki Forest virus membrane glycoproteins. *J. Cell Sci.* 102 505–513. 1506431 10.1242/jcs.102.3.505

[B32] LaGrassa T. J. Ungermann C. (2005). The vacuolar kinase Yck3 maintains organelle fragmentation by regulating the HOPS tethering complex. *J. Cell Biol.* 168 401–414. 10.1083/jcb.200407141 15684030 PMC2171739

[B33] Li S. C. Kane P. M. (2009). The yeast lysosome-like vacuole: endpoint and crossroads. *Biochim Biophys Acta* 1793 650–663. 10.1016/j.bbamcr.2008.08.003 18786576 PMC2906225

[B34] Liu W. Xie S. Zhao X. Chen X. Zheng W. Lu G. (2010). A homeobox gene is essential for conidiogenesis of the rice blast fungus *Magnaporthe oryzae*. *Mol. Plant Microbe Interact.* 23 366–375. 10.1094/MPMI-23-4-0366 20192824

[B35] Liu X. Hu G. Panepinto J. Williamson P. R. (2006). Role of a VPS41 homologue in starvation response, intracellular survival and virulence of *Cryptococcus neoformans*. *Mol. Microbiol.* 61 1132–1146. 10.1111/j.1365-2958.2006.05299.x 16879414

[B36] Marcel S. Sawers R. Oakeley E. Angliker H. Paszkowski U. (2010). Tissue-adapted invasion strategies of the rice blast fungus *Magnaporthe oryzae*. *Plant Cell* 22 3177–3187. 10.1105/tpc.110.078048 20858844 PMC2965542

[B37] Matile P. (1978). Biochemistry and function of vacuoles. *Ann. Rev. Plant Physiol.* 29 193–213. 10.1146/annurev.pp.29.060178.001205

[B38] Medvedev R. Hildt E. Ploen D. (2016). Look who’s talking—the crosstalk between oxidative stress and autophagy supports exosomal-dependent release of HCV particles. *Cell Biol. Toxicol.* 27 382–393. 10.1007/s10565-016-9376-3 27987184

[B39] Menke J. R. (2011). *A Study of Fusarium graminearum Virulence Factors.* Minneapolis, MN: University of Minnesota.

[B40] Mentlak T. A. Kombrink A. Shinya T. Ryder L. S. Otomo I. Saitoh H. (2012). Effector-mediated suppression of chitin-triggered immunity by *Magnaporthe oryzae* is necessary for rice blast disease. *Plant Cell* 24 322–335. 10.1105/tpc.111.092957 22267486 PMC3289562

[B41] Möst T. (2014). *Salmonella virulence Factors and Their Role in Intracellular Parasitism.* The Ph.D. thesis, Aix-Marseille Universite Marseille.

[B42] Nesher I. Barhoom S. Sharon A. (2008). Cell cycle and cell death are not necessary for appressorium formation and plant infection in the fungal plant pathogen *Colletotrichum gloeosporioides*. *BMC Biol.* 6:9 10.1186/1741-7007-6-9 PMC2276476 18275611

[B43] Nickerson D. P. Brett C. L. Merz A. J. (2009). Vps-C complexes: gatekeepers of endolysosomal traffic. *Curr. Opin. Cell Biol.* 21 543–551. 10.1016/j.ceb.2009.05.007 19577915 PMC2807627

[B44] Ohneda M. Arioka M. Nakajima H. Kitamoto K. (2002). Visualization of vacuoles in *Aspergillus oryzae* by expression of CPY-EGFP. *Fungal Genet. Biol.* 37 29–38. 10.1016/S1087-1845(02)00033-6 12223187

[B45] Pereira C. Pereira S. Pissarra J. (2014). Delivering of proteins to the plant vacuole—an update. *Int. J. Mol. Sci.* 15 7611–7623. 10.3390/ijms15057611 24802873 PMC4057694

[B46] Petre B. Kamoun S. (2014). How do filamentous pathogens deliver effector proteins into plant cells? *PLoS Biol.* 12:e1001801 10.1371/journal.pbio.1001801 PMC3934835 24586116

[B47] Pollack J. K. Harris S. D. Marten M. R. (2009). Autophagy in filamentous fungi. *Fungal Genet. Biol.* 46 1–8. 10.1016/j.fgb.2008.10.010 19010432

[B48] Radisky D. C. Snyder W. B. Emr S. D. Kaplan J. (1997). Characterization of VPS41 a gene required for vacuolar trafficking and high-affinity iron transport in yeast. *Proc. Natl. Acad. Sci. U.S.A*. 94 5662–5666. 10.1073/pnas.94.11.5662 9159129 PMC20835

[B49] Ramanujam R. Calvert M. E. Selvaraj P. Naqvi N. I. (2013). The late endosomal HOPS complex anchors active G-protein signaling essential for pathogenesis in *Magnaporthe oryzae*. *PLoS Pathog.* 9:e1003527 10.1371/journal.ppat.1003527 PMC3731250 23935502

[B50] Ramsay L. M. Gadd G. M. (1997). Mutants of *Saccharomyces cerevisiae* defective in vacuolar function confirm a role for the vacuole in toxic metal ion detoxification. *FEMS Microbiol. Lett.* 152 293–298. 10.1111/j.1574-6968.1997.tb10442.x 9231423

[B51] Raymond C. K. Howald-Stevenson I. Vater C. Stevens T. (1992). Morphological classification of the yeast vacuolar protein sorting mutants: evidence for a prevacuolar compartment in class E vps mutants. *Mol. Biol. Cell* 3 1389–1402. 10.1091/mbc.3.12.1389 1493335 PMC275707

[B52] Richards A. Gow N. A. Veses V. (2012). Identification of vacuole defects in fungi. *J. Microbiol. Methods* 91 155–163. 10.1016/j.mimet.2012.08.002 22902527

[B53] Runkle K. B. (2012). *Bif-1 Regulates EGFR Endocytosis and Chemotactic Cell Migration in Breast Cancer.* Doctor of Philosophy, The Pennsylvania State University State College, PA.

[B54] Sato T. K. Rehling P. Peterson M. R. Emr S. D. (2000). Class C Vps protein complex regulates vacuolar SNARE pairing and is required for vesicle docking/fusion. *Mol. Cell* 6 661–671. 10.1016/S1097-2765(00)00064-2 11030345

[B55] Schröter S. Beckmann S. Schmitt H. D. (2016). Coat/tether interactions—exception or rule? *Front. Cell Dev. Biol.* 4:44 10.3389/fcell.2016.00044 PMC4868844 27243008

[B56] Smith L. M. (2015). *Investigating Phagosome Dynamics of Microbial Pathogens.* Ph.D. thesis, University of Birmingham Birmingham.

[B57] Soanes D. M. Alam I. Cornell M. Wong H. M. Hedeler C. Paton N. W. (2008). Comparative genome analysis of filamentous fungi reveals gene family expansions associated with fungal pathogenesis. *PLoS ONE* 3:e2300 10.1371/journal.pone.0002300 PMC2409186 18523684

[B58] Soanes D. M. Richards T. A. Talbot N. J. (2007). Insights from sequencing fungal and oomycete genomes: what can we learn about plant disease and the evolution of pathogenicity? *Plant Cell* 19 3318–3326. 10.1105/tpc.107.056663 18024565 PMC2174898

[B59] Spang A. (2016). Membrane tethering complexes in the endosomal system. *Front. Cell Dev. Biol.* 4:35 10.3389/fcell.2016.00035 PMC4860415 27243003

[B60] Stachowiak J. C. Brodsky F. M. Miller E. A. (2013). A cost-benefit analysis of the physical mechanisms of membrane curvature. *Nat. Cell Biol.* 15 1019–1027. 10.1038/ncb2832 23999615 PMC3813008

[B61] Talbot N. J. (2003). On the trail of a serial killer: exploring the biology of *Magnaporthe grisea*. *Annu. Rev. Microbiol.* 57 177–202. 10.1146/annurev.micro.57.030502.090957 14527276

[B62] Talbot N. J. Wilson R. A. (2009). Under pressure: investigating the biology of plant infection by *Magnaporthe oryzae*. *Nat. Rev. Microbiol.* 7 185–195. 10.1038/nrmicro2032 19219052

[B63] Teh O.-K. Hofius D. (2014). Membrane trafficking and autophagy in pathogen-triggered cell death and immunity. *J. Exp. Bot.* 65 1297–1312. 10.1093/jxb/ert441 24420567

[B64] Thomma B. P. Nürnberger T. Joosten M. H. (2011). Of PAMPs and effectors: the blurred PTI-ETI dichotomy. *Plant Cell* 23 4–15. 10.1105/tpc.110.082602 21278123 PMC3051239

[B65] Veses V. Richards A. Gow N. A. (2009). Vacuole inheritance regulates cell size and branching frequency of *Candida albicans* hyphae. *Mol. Microbiol.* 71 505–519. 10.1111/j.1365-2958.2008.06545.x 19040629 PMC2680324

[B66] Wang C.-M. Xu W. Liu J. Zhang J.-G. Saraf L. V. Arey B. W. (2011). In situ transmission electron microscopy observation of microstructure and phase evolution in a SnO2 nanowire during lithium intercalation. *Nano Lett.* 11 1874–1880. 10.1021/nl200272n 21476583

[B67] Wang J. Sheppard G. S. Lou P. Kawai M. Park C. Egan D. A. (2003). Physiologically relevant metal cofactor for methionine aminopeptidase-2 is manganese. *Biochemistry* 42 5035–5042. 10.1021/bi020670c 12718546

[B68] Wang Y. Pang J. Zheng Y. Jiang P. Gong W. Chen X. (2017). Genetic manipulation of the bifunctional gene, carRA, to enhance lycopene content in Blakeslea trispora. *Biochem. Eng. J.* 119 27–33. 10.1016/j.bej.2016.12.011

[B69] Wetherbee R. Andersen R. A. Pickett-Heaps J. D. (2012). *The Protistan Cell Surface.* Berlin: Springer Science & Business Media.

[B70] Wong D. Chao J. D. Av-Gay Y. (2013). *Mycobacterium tuberculosis*-secreted phosphatases: from pathogenesis to targets for TB drug development. *Trends Microbiol.* 21 100–109. 10.1016/j.tim.2012.09.002 23084287

[B71] Yan X. Li Y. Yue X. Wang C. Que Y. Kong D. (2011). Two novel transcriptional regulators are essential for infection-related morphogenesis and pathogenicity of the rice blast fungus *Magnaporthe oryzae*. *PLoS Pathog.* 7:e1002385 10.1371/journal.ppat.1002385 PMC3228794 22144889

[B72] Yan X. Talbot N. J. (2016). Investigating the cell biology of plant infection by the rice blast fungus Magnaporthe oryzae. *Curr. Opin. Microbiol.* 34 147–153. 10.1016/j.mib.2016.10.001 27816794

[B73] Yang X. Cui H. Cheng J. Xie J. Jiang D. Hsiang T. (2016). A HOPS protein, CmVps39 is required for vacuolar morphology, autophagy, growth, conidiogenesis and mycoparasitic functions of *Coniothyrium minitans*. *Environ. Microbiol.* 18 3785–3797. 10.1111/1462-2920.13334 27105005

[B74] Yoshida K. Saunders D. G. Mitsuoka C. Natsume S. Kosugi S. Saitoh H. (2016). Host specialization of the blast fungus *Magnaporthe oryzae* is associated with dynamic gain and loss of genes linked to transposable elements. *BMC Genomics* 17:370 10.1186/s12864-016-2690-6 PMC4870811 27194050

[B75] Yu I. M. Hughson F. M. (2010). Tethering factors as organizers of intracellular vesicular traffic. *Annu. Rev. Cell Dev. Biol.* 26 137–156. 10.1146/annurev.cellbio.042308.113327 19575650

[B76] Zhang C. Zong H. Zhuge B. Lu X. Fang H. Zhu J. (2016). Protoplast preparation and polyethylene glycol (PEG)-mediated transformation of *Candida glycerinogenes*. *Biotechnol. Bioprocess Eng.* 21 95–102. 10.1007/s12257-015-0686-8

[B77] Zheng H. Chen S. Chen X. Liu S. Dang X. Yang C. (2016). The small GTPase MoSec4 Is involved in vegetative development and pathogenicity by regulating the extracellular protein secretion in *Magnaporthe oryzae*. *Front. Plant Sci.* 7:1458. 10.3389/fpls.2016.01458 PMC5037964 27729922

[B78] Zheng H. Zheng W. Wu C. Yang J. Xi Y. Xie Q. (2015). Rab GTPases are essential for membrane trafficking-dependent growth and pathogenicity in *Fusarium graminearum*. *Environ. Microbiol.* 17 4580–4599. 10.1111/1462-2920.12982 26177389

[B79] Zheng W. Zhao X. Xie Q. Huang Q. Zhang C. Zhai H. (2012). A conserved homeobox transcription factor Htf1 is required for phialide development and conidiogenesis in *Fusarium* species. *PLoS ONE* 7:e45432 10.1371/journal.pone.0045432 PMC3448628 23029006

[B80] Zheng W. Zhou J. He Y. Xie Q. Chen A. Zheng H. (2015). Retromer is essential for autophagy-dependent plant infection by the rice blast fungus. *PLoS Genet.* 11:e1005704 10.1371/journal.pgen.1005704 PMC4686016 26658729

[B81] Zhong Z. Norvienyeku J. Yu J. Chen M. Cai R. Hong Y. (2015). Two different subcellular-localized Acetoacetyl-CoA acetyltransferases differentiate diverse functions in *Magnaporthe oryzae*. *Fungal Genet. Biol.* 83 58–67. 10.1016/j.fgb.2015.08.008 26318870

