# Supplementary material for: A HOPS Protein, MoVps41, Is Crucially Important for Vacuolar Morphogenesis, Vegetative Growth, Reproduction and Virulence in Magnaporthe oryzae
Source: Front Plant Sci. 2017 Jun 30;8:1091. doi: 10.3389/fpls.2017.01091 (PMC5492488; doi:10.3389/fpls.2017.01091)
Supplement: Supplementary file 1 [file Data_Sheet_1.PDF]

Supporting Information

Supplementary figure1. Domain analysis conducted by SMART and Pfam, and domain figures were drawn by IBS. The sequences evaluated in the domain analysis were from *Magnaporthe oryzae* (MoVps41), *Saccharomyces cerevisiae* (NP_010365.3), *Fusarium graminearum* (XP_011324698.1), *Arobidopsis thaliana* (NP_172297.2), *Mus musculus* (EDL32708.1), and *Homo sapiens* (NP_055211.2), All these proteins possess the six conserved clathrin domains of vacuolar protein sorting.

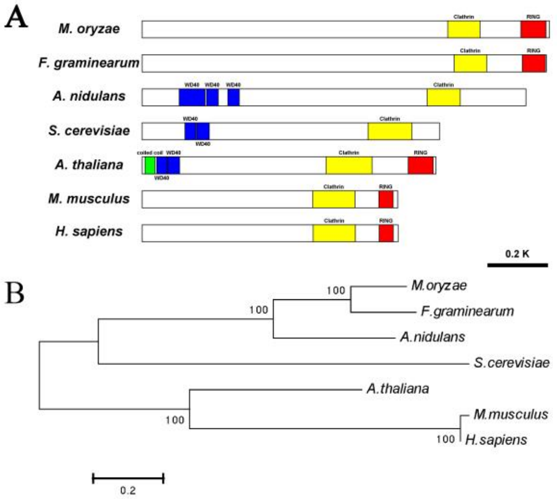

Table S1. Primers used in this study

Name	Sequence(5'-3')
VPS41-AF	5'AGGCTGTCTGACTGTTTCG3'
VPS41-AR	5'TTGACCTCCACTAGCTCCAGCCAAGCCCACCAGGTCCTTCCGTATC3'
VPS41-BF	5'GAATAGAGTAGATGCCGACCGCGGGTTAGCCGCAGTAGACATTCC3'
VPS41-BR	5'TTGCTGGTTCAGACGACA3'
HYG/F	5'GGCTTGGCTGGAGCTAGTGGAGGTCAA3'
HY/R	5'GTATTGACCGATTCCTTGCGGTCCGAA3'
YG/F	5'GATGTAGGAGGGCGTGGATATGTCCT3'

HYG/R	5'AACCCGCGGTCGGCATCTACTCTATTC3'
VPS41-OF	5'GAGACTCCCAATGTCAATAACG3'
VPS41-OR	5'TCTGCCCACGGACTTTTC3'
VPS41-UA	5'TGCTGCGGGAGTCTATTC3'
H853	5'GACAGACGTCGCGGTGAGTT3'
VPS41-com-F	5'AGAAGTAGGTGCCTCCAAGAAGC3'
VPS41-com-R	5'GGTTTCTTACCACCACTCTTGC3'
VPS41-GF	5'AAAGCTGGGTACCGGGCCCCCCAGAAGTAGGTGCCTCCAAGAAGC3'
VPS41-GR	5'GTGTCGACCTGCAGGCATGCCATTTCCTTACTCCTATCAC3'

Table S2. Sequence alignments between MoVps41 and its homologs.

Species	Full length	CHCR domain	RING-H2 motif
F. graminearum	62%	72%	53%
A. nidulans	46%	64%	No
S. cerevisiae	23%	38%	No
A. thaliana	25%	38%	31%
M. musculus	27%	45%	50%
H. sapiens	27%	45%	50%
