# Supplementary material for: A HOPS Protein, MoVps41, Is Crucially Important for Vacuolar Morphogenesis, Vegetative Growth, Reproduction and Virulence in Magnaporthe oryzae
Source: Front Plant Sci. 2017 Jun 30;8:1091. doi: 10.3389/fpls.2017.01091 (PMC5492488; doi:10.3389/fpls.2017.01091)
Supplement: Supplementary file 2 [file Data_Sheet_2.PDF]

```
1 M.oryzae          1   ..MSD...EETPNVNNGIQSIGDYTESSPQT..PSQPPNVAVSDERAAESTLEKVAS...
2 F.graminearum     1   ..MTDQSPEPSHEQARDLETQQPEHDAAPTSSEPVPPTNNDASDDGAHSSESESISKEGD
3 A.nidulans        1   ...MDAPGATEASLSRVEPGQGSPTRPSTHHRRPHRQLHTETGDDDEEEQE.........
4 S.cerevisiae      1   MTTDNHQNDSVLDQQSGERTIDESNSISDENNVDNKREDVNVTSPTKSVSCISQAENG..
5 A.thaliana        1   ......................................MAAVPPENGV............
6 M.musculus            ............................................................
7 H.sapiens             ............................................................

1 M.oryzae         51   ......TP........................DEDGDESEEEEDDEDDDDEDEEDDEEE.
2 F.graminearum    59   VSAKDVTPPGSNDGKASQDGDQDKEGGEEDDNDEDDDNDEEDDDDEDEDDEEDEDEEEED
3 A.nidulans       49   ................................DPNREREESMTETMSYYGEEEEDDEEE.
4 S.cerevisiae     59   ......................VASRTDESTITGSATDAETGDDDDDDDDDDDEDEDDE.
5 A.thaliana       11   ................................DGDDEREEEEEDEEEEEEEEEEENGDE.
6 M.musculus        1   ....................................MAEAEEQETESLEESTDESEEE..
7 H.sapiens         1   ....................................MAEAEEQETGSLEESTDESEEEE.

1 M.oryzae         80   ........EEPRLKYTRLTQNLGAVYKNGDATSSFLVAGDKMIIGTHSGNIHAVQLPSFQ
2 F.graminearum   119   EEDEEDDDEEPKLKYARLTQHLNGVYRNGDATSAFLVAGDKMIVGTHNGNIHIVQLPMFQ
3 A.nidulans       76   ........EEPHLKCSHLTKQLGSAYRNGDATSTFLAAGDKMVLGTHNGNIHVFSVPLFR
4 S.cerevisiae     96   .......DEPPLLKYTRISQLPKNFFQR.DSISSCLFGDTFFAFGTHSG.ILHLTTCAFE
5 A.thaliana       38   ......AEEEPRLKYQRMGGNVPALLSN.DAASCIAVAARMIALGTHDGTVRILDLLGN.
6 M.musculus       23   ......SEEEPKLKYERLSNGVTEILQK.DAASCMTVHDKFLALGTHYGKVYLLDVQGN.
7 H.sapiens        24   ......SEEEPKLKYERLSNGVTEILQK.DAASCMTVHDKFLALGTHYGKVYLLDVQGN.

1 M.oryzae        132   TLLVYHAHSASVTSVSISPCPPPVSSLRPDVLKAAPQNSETASLRRPASKHSEASPAAAS
2 F.graminearum   179   SMRVYHAHSASVTSISISPYPPPLP...TDIPETVSKHGAHSNASISSSRQTDSSSPAPS
3 A.nidulans      128   SLRVYHAHSATVTSISISPFPPPAPNLKHELATRFAENQSASTKPFSSS........GSI
4 S.cerevisiae    147   PIKTIKCHRSSILCINTD..........................................
5 A.thaliana       90   QVKEFRAHTAPVNDINFD..........................................
6 M.musculus       75   ITQKFDVSPVKINQISLD..........................................
7 H.sapiens        76   ITQKFDVSPVKINQISLD..........................................

1 M.oryzae        192   RKPREAPPVPNMPSNHIHIATSSMDGNVCVQSLMERRDVQLRNFARPVQAVALSPEYK..
2 F.graminearum   236   RRPREVSQIPRTPSNDIYVATSSLDGNVCVQNLIDMKDVQLRNFARPVQTVALSPEFK..
3 A.nidulans      180   RGQSKPTSLPTTPSNSIYIASSSIDGNVCVASLLDTKDVLLRNFGRPVQAVALSPEYK..
4 S.cerevisiae    165   ...............GKYFATGSIDGTVIIGSMDDPQNITQYDFKRPINSVALHSNFQ..
5 A.thaliana      108   .............TEGEYIGSCSDDGSVVINSLFTDDEKMKFDYHRPMKAISLDPDYTKK
6 M.musculus       93   .............DSGEHMGVCSEDGKLQVFGLYSGEE.FHETFDCPIKIIAVHPQFVRS
7 H.sapiens        94   .............ESGEHMGVCSEDGKVQVFGLYSGEE.FHETFDCPIKIIAVHPHFVRS

1 M.oryzae        250   SDKTYLSGGLAGSLILTVGGQTGKSN.STTIGTAAATASSWLGSIGLAGNAGKDTVLHSG
2 F.graminearum   294   TDRTYLSGGLAGQLILTAGGGPGRST.STTTGTAAATASGWLGSMGLGGNAGKDTILHSG
3 A.nidulans      238   SDRTFISGGRAGELILTTGGKVGVSTNSTTLGGAAATASSWLGTFGLGGNTGKDTVLHSG
4 S.cerevisiae    208   ASRMFVSGGMAGDVVLSQRNWLG.............NRIDIVLNKKKKKKTRKDDLSSDM
5 A.thaliana      155   QSKRFVAGGLAGHLYMNSKK...........................WFG.NKDQVLHSG
6 M.musculus      139   SCKQFVTGGKK..LLLFERT............................WMNRWKSSVLHEG
7 H.sapiens       140   SCKQFVTGGKK..LLLFERS............................WMNRWKSAVLHEG

1 M.oryzae        309   EGTISTIKWSLSGKYVAWLNEHGIKIMRSKIKLDSADADDAWKRIGHIDRPQSSEWDAMA
2 F.graminearum   353   EGTISTIKWSLSGKYVVWLNEHGIKIMRSKLHLESADAEDAWKRVGHIDRPQTDEWETMA
3 A.nidulans      298   EGAISTVKWSLSGKYVAWVNEEGIKIMRSNLHLDSVDSELAWKRVSHTDRPNRQGWEEMA
4 S.cerevisiae    255   KGPIMGIYTM..GDLILWMDDDGITFCDVPTRSQLLN........IPFPSRIFNVQDVRP
5 A.thaliana      187   EGPIHSVKWR..GSLIAWANDVGVKVYDTAKDQR.............VTFIEKPRGSPRP
6 M.musculus      170   EGNIRSVKWR..GHLIAWANNMGVKVFDITSKQR.............ISNVPRDDISLRP
7 H.sapiens       171   EGNIRSVKWR..GHLIAWANNMGVKIFDIISKQR.............ITNVPRDDISLRP
```

```
1 M.oryzae        369  SAWRGRAEWMDEQAVEMDDNADTTLVGDSRSDQDSSGTLSPAAAKLKQQQSKHDKTIEKL
2 F.graminearum   413  SVWKGRVEWIDEQAVEVDETDQSATEKSS..........SPATEKLKGHALASKKGIERL
3 A.nidulans      358  SVWKARAEWVDESLLESEHLTSNNTGGEAS.........TPTQSTIIKERQ......EKL
4 S.cerevisiae    305  DLFRPHVHFLES.............................................DRV
5 A.thaliana      232  EALLPHLVWQDD.............................................TLL
6 M.musculus      215  DMYPCSLCWKDN.............................................VTL
7 H.sapiens       216  DMYPCSLCWKDN.............................................VTL

1 M.oryzae        429  VVGWGGTIWVIHVHPGGMGVGKNAGEKSVG..............................
2 F.graminearum   463  LVGWGGTIWIIHVHPGGVGVGRHAGEKTIG..............................
3 A.nidulans      403  VVGWGGTVWVINVYPD..RPNKSNRDLRIG..............................
4 S.cerevisiae    320  VIGWGSNIWLFKVSFTKDSNSIKSGDSNSQSNNMSHFNPTTNIGSLLSSAASSFRGTPDK
5 A.thaliana      247  VIGWGTSVKIASIKSDQQQTGTFRQIQMSSLT............................
6 M.musculus      230  IIGWGTSIKICSVK..ERHASEMRDLPSR...............................
7 H.sapiens       231  IIGWGTSVKVCSVK..ERHASEMRDLPSR...............................

1 M.oryzae        459  RAEIHQKLRMDCIISGISLYTQSLLLILAYCLPEDEDEDDDEDGDGEKTPTQPRGHRSRP
2 F.graminearum   493  RAEIVKILRMDCIISGISLYTQNLLLVLAYCLPEDDDED.....EGAVSVSNSPDKRHKP
3 A.nidulans      431  SVEVATILRTDCIISGISLYSPSHLVVLAYIEAENEPAD.....................
4 S.cerevisiae    380  KVELECHFTVSMLITGLASFKDDQLLCLGFDIDIEEEATIDEDMKEGKNFS.........
5 A.thaliana      279  QVDIVASFQTSYYISGIAPFGDSLVILAYIPIEGDGEKEFSS..................
6 M.musculus      257  YVEIVSQFETEFYISGLAPLCDQLVVLSYV.......KEVSE..................
7 H.sapiens       258  YVEIVSQFETEFYISGLAPLCDQLVVLSYV.......KEISE..................

1 M.oryzae        519  SAASSSSEPSGGIRRRQNNQPPELRLIDLKSQSEIYKDGLSISRYERLSASDYHLGVLPA
2 F.graminearum   548  .NSSTGSQPSGGLPRRQNNQPPELRLIDLNSQAEADKDSLSVSRYERLSSGDYHLGVLPA
3 A.nidulans      470  .........EQITRRRPRGQEPELRIIDIESKEEVSADTLAISRYENLTASDYHMSVLPP
4 S.cerevisiae    431  .........KRPENLLAKGNAPELKIVDLFNGDEIYNDEVIMKNYEKLSINDYHLGKH..
5 A.thaliana      321  .........TTTLSRQGNAQRPEIRIVSWNN.DELTMDALPVHGFEHYKAKDYSLAHAPF
6 M.musculus      292  .........KTEREYCARPRLDIIQPLPETC.EEISSDALTVRGFQENECRDYHLEYS..
7 H.sapiens       293  .........KTEREYCARPRLDIIQPLSETC.EEISSDALTVRGFQENECRDYHLEYS..

1 M.oryzae        579  QNQE.AVASSKSALEALAGFGTDMWNAAIKPKMLFSSGASIRSGHS.NDATSGSKVSLAG
2 F.graminearum   607  RNAASAIASSRGALEAIAGIGTDMWNAAINPRSLFSSGASIRSRGS.GDDSS.IRGSTAG
3 A.nidulans      521  WKTN.MPVSQRGALEALG...TGLWDATMYPARLFSSAASIRSSTSSGDRSSNRAPSTFA
4 S.cerevisiae         ............................................................
5 A.thaliana      371  PGSS........................................................
6 M.musculus           ............................................................
7 H.sapiens            ............................................................

1 M.oryzae        637  SSIRAPFKLQTQAVHPSLETPGPKIFMHSPYDCVLATKRDLGDHLTWLVEHQEYQAAWEL
2 F.graminearum   665  TIRPGTSRGLSPTVHSGLVKPGVKIFIHSPYDCILATRRDLSDHLGWLLERQQYQRAWEL
3 A.nidulans      577  SRRSVPEEPLAIEVQEVAESSGPKIFVHSPYDCVAALKRDLADHLAWLIEHEKYAEAWKL
4 S.cerevisiae    480  .................IDKTTPEYYLISSNDAIRVQELSLKDHFDWFMERKQYYKAWKI
5 A.thaliana      375  ...........YAGGQWAAGDEPLYYIVSPKDVVIAKPRDAEDHINWLLQHGFHEKA...
6 M.musculus      340  ...................EGESLFYVVSPRDVVVAKERDQDDHIDWLLEKKKYEEA...
7 H.sapiens       341  ...................EGESLFYIVSPRDVVVAKERDQDDHIDWLLEKKKYEEA...

1 M.oryzae        697  LDEHPEIMATSPSVSDLVATPTAERPTQVSDDLFDDTTSTVDAVSRAFNSSVAREKRRIG
2 F.graminearum   725  LDEHPEIMAPAGERANDNTPPTPTLNQEASDEFNDDE.SVIDSQVRDFYSSAEKEKRRIG
3 A.nidulans      637  LDEHPEAAGSS..EGSDNVSITPGRSQTSLGDLFVDDRSSITATDRGNVPAAVQEKRRIG
4 S.cerevisiae    523  G...............................KYVIGSEERFSIGLKFLNSLVTKKDWGT
5 A.thaliana      421  ........................................LAAVEASEG..RTELIDKVG
6 M.musculus      378  ........................................LMAAEISQRNIKRHKILDIG
7 H.sapiens       379  ........................................LMAAEISQKNIKRHKILDIG
```

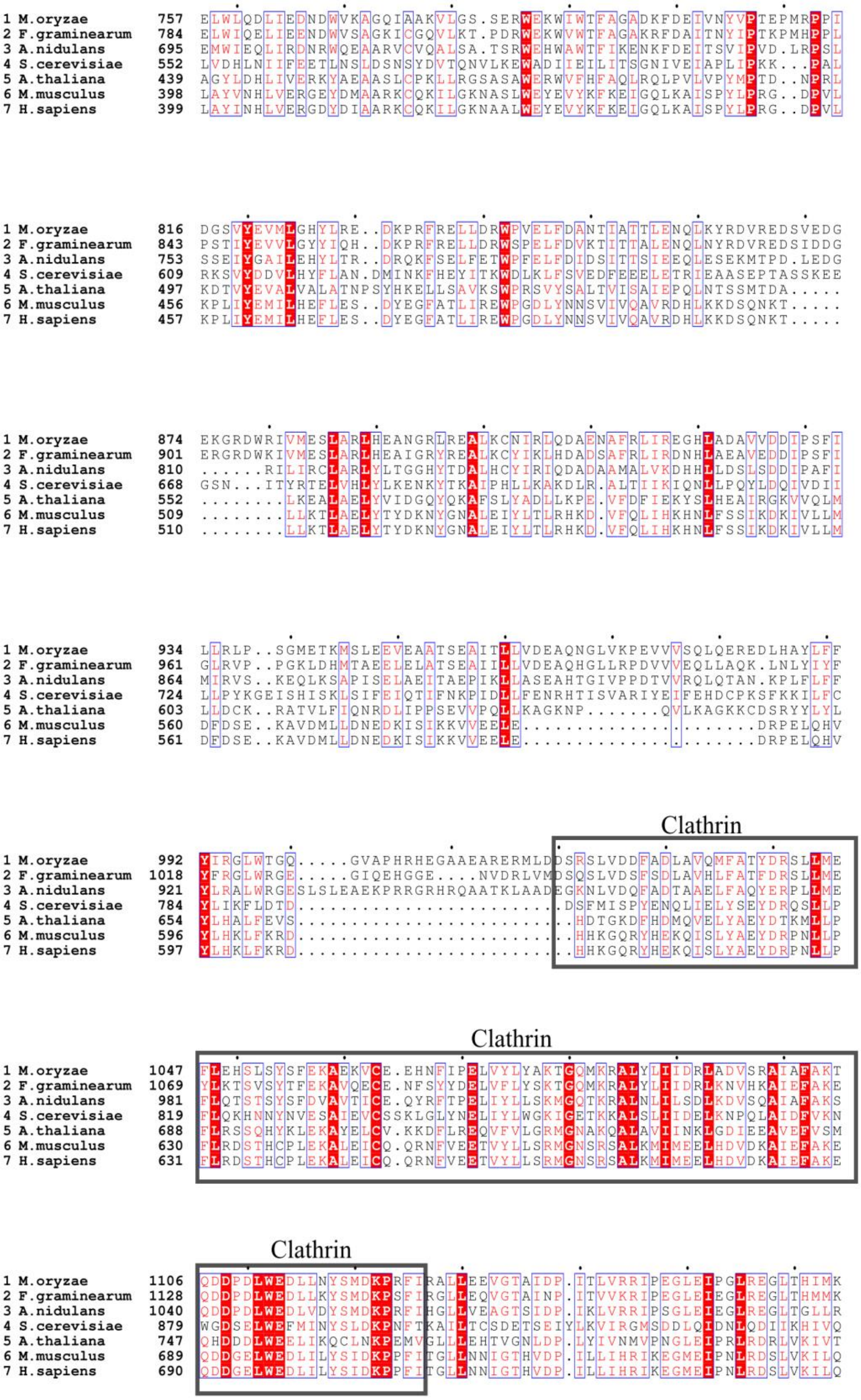
1 M.oryzae 757 ELWLQDLIEDNDWVKAGQIAAKVLGS.SERWEKWIWTFAGADKFDEIVNYVPTEPMRPPI
2 F.graminearum 784 ELWIQELIEENDWVSAGKICGQVLKT.PDRWEKWVWTFAGAKRFDAITNYIPTKPMHPPL
3 A.nidulans 695 EMWIEQLIRDNRWQEAARVCVQALSA.TSRWEHWAWTFIKENKFDEITSVIPVD.LRPSL
4 S.cerevisiae 552 LVDHLNIIFEETLNSLDSNSYDVTQNVLKEWADIIEILITSGNIVEIAPLIPKK...PAL
5 A.thaliana 439 AGYLDHLIVERKYAEAASLCPKLLRGSASAWERWVFHFAQLRQLPVLVPYMPTD..NPRL
6 M.musculus 398 LAYVNHLVERGEYDMAARKCQKILGKNASLWEYEVYKFKEIGQLKAISPYLPRG..DPVL
7 H.sapiens 399 LAYINHLVERGDYDIAARKCQKILGKNAALWEYEVYKFKEIGQLKAISPYLPRG..DPVL
1 M.oryzae 816 DGSVYEVMLGHYLRE..DKPRFRELLDRWPVELFDANTIATTLENQLKYRDVREDSVEDG
2 F.graminearum 843 PSTIYEVVLGYYIQH..DKPRFRELLDRWSPELFDVKTITTALENQLNYRDVREDSIDDG
3 A.nidulans 753 SSEIYGAILEHYLTR..DRQKFSELFETWPFELFDIDSITTSIEEQLESEKMTPD.LEDG
4 S.cerevisiae 609 RKSVYDDVLHYFLAN.DMINKFHEYITKWDLKLFSVEDFEEELETRIEAASEPTASSKEE
5 A.thaliana 497 KDTVYEVALVALATNPSYHKELLSAVKSWPRSVYSALTVISAIEPQLNTSSMTDA.....
6 M.musculus 456 KPLIYEMILHEFLES..DYEGFATLIREWPGDLYNNSVIVQAVRDHLKKDSQNKT.....
7 H.sapiens 457 KPLIYEMILHEFLES..DYEGFATLIREWPGDLYNNSVIVQAVRDHLKKDSQNKT.....
1 M.oryzae 874 EKGRDWRIVMESLARLHEANGRLREALKCNIRLQDAENAFRLIREGHLADAVVDDIPSFI
2 F.graminearum 901 ERGRDWKIVMESLARLHEAIGRYREALKCYIKLHDADSAFRLIRDNHLAEAVEDDIPSFI
3 A.nidulans 810 ......RILIRCLARLYLTGGHYTDALHCYIRIQDADAAMALVKDHHLLDSLSDDIPAFI
4 S.cerevisiae 668 GSN...ITYRTELVHLYLKENKYTKAIPHLLKAKDLR.ALTIIKIQNLLPQYLDQIVDII
5 A.thaliana 552 ........LKEALAELYVIDGQYQKAFSLYADLLKPE.VFDFIEKYSLHEAIRGKVVQLM
6 M.musculus 509 ........LLKTLAELYTYDKNYGNALEIYLTLRHKD.VFQLIHKHNLFSSIKDKIVLLM
7 H.sapiens 510 ........LLKTLAELYTYDKNYGNALEIYLTLRHKD.VFQLIHKHNLFSSIKDKIVLLM
1 M.oryzae 934 LLRLP..SGMETKMSLEEVEAATSEAITLLVDEAQNGLVKPEVVVSQLQEREDLHAYLFF
2 F.graminearum 961 GLRVP..PGKLDHMTAEELELATSEAIILLVDEAQHGLLRPDVVVEQLLAQK.LNLYIYF
3 A.nidulans 864 MIRVS..KEQLKSAPISELAEITAEPIKLLASEAHTGIVPPDTVVRQLQTAN.KPLFLFF
4 S.cerevisiae 724 LLPYKGEISHISKLSIFEIQTIFNKPIDLLFENRHTISVARIYEIFEHDCPKSFKKILFC
5 A.thaliana 603 LLDCK..RATVLFIQNRDLIPPSEVVPQLLKAGKNP.......QVLKAGKKCDSRYYLYL
6 M.musculus 560 DFDSE..KAVDMLLDNEDKISIKKVVEELE......................DRPELQHV
7 H.sapiens 561 DFDSE..KAVDMLLDNEDKISIKKVVEELE......................DRPELQHV
Clathrin
1 M.oryzae 992 YIRGLWTGQ.....GVAPHRHEGAAEARERMLDDSRSLVDDFADLAVQMFATYDRSLLME
2 F.graminearum 1018 YFRGLWRGE.....GIQEHGGE....NVDRLVMDSQSLVDSFSDLAVHLFATFDRSLLME
3 A.nidulans 921 YLRALWRGESLSLEAEKPRRGRHRQAATKLAADEGKNLVDQFADTAAELFAQYERPLLME
4 S.cerevisiae 784 YLIKFLDTD.........................DSFMISPYENQLIELYSEYDRQSLLP
5 A.thaliana 654 YLHALFEVS..........................HDTGKDFHDMQVELYAEYDTKMLLP
6 M.musculus 596 YLHKLFKRD..........................HHKGQRYHEKQISLYAEYDRPNLLP
7 H.sapiens 597 YLHKLFKRD..........................HHKGQRYHEKQISLYAEYDRPNLLP
Clathrin
1 M.oryzae 1047 FLEHSLSYSFEKAEKVCE.EHNFIPELVYLYAKTGQMKRALYLIIDRLADVSRAIAFAKT
2 F.graminearum 1069 YLKTSVSYTFEKAVQECE.NFSYYDELVFLYSKTGQMKRALYLIIDRLKNVHKAIEFAKE
3 A.nidulans 981 FLQTSTSYSFDVAVTICE.QYRFTPELIYLLSKMGQTKRALNLIILSDLKDVSQAIAFAKS
4 S.cerevisiae 819 FLQKHNNYNVESAIEVCSSKLGLYNELIYLWGKIGETKKALSLIIDELKNPQLAIDFVKN
5 A.thaliana 688 FLRSSQHYKLEKAYELCV.KKDFLREQVFVLGRMGNAKQALAVIINKLGDIEEAVEFVSM
6 M.musculus 630 FLRDSTHCPLEKALEICQ.QRNFVEETVYLLSRMGNSRSALKMIMEELHDVDKAIEFAKE
7 H.sapiens 631 FLRDSTHCPLEKALEICQ.QRNFVEETVYLLSRMGNSRSALKMIMEELHDVDKAIEFAKE
Clathrin
1 M.oryzae 1106 QDDPDLWEDLLNYSMDKPRFIRALLEEVGTAIDP.ITLVRRIPEGLEIPGLREGLTHIMK
2 F.graminearum 1128 QDDPDLWEDLLKYSMDKPSFIRGLLEQVGTAINP.ITVVKRIPEGLEIEGLREGLTHMMK
3 A.nidulans 1040 QDDPDLWEDLVDYSMDKPRFIHGLLVEAGTSIDP.IKLVRRIPSGLEIEGLREGLTGLLR
4 S.cerevisiae 879 WGDSELWEFMINYSLDKPNFTKAILTCSDETSEIYLKVIRGMSDDLQIDNLQDIIKHIVQ
5 A.thaliana 747 QHDDDLWEELIKQCLNKPEMVGLLLEHTVGNLDP.LYIVNMVPNGLEIPRLRDRLVKIVT
6 M.musculus 689 QDDGELWEDLILYSIDKPPFITGLLNNIGTHVDP.ILLIHRIKEGMEIPNLRDSLVKILQ
7 H.sapiens 690 QDDGELWEDLILYSIDKPPFITGLLNNIGTHVDP.ILLIHRIKEGMEIPNLRDSLVKILQ

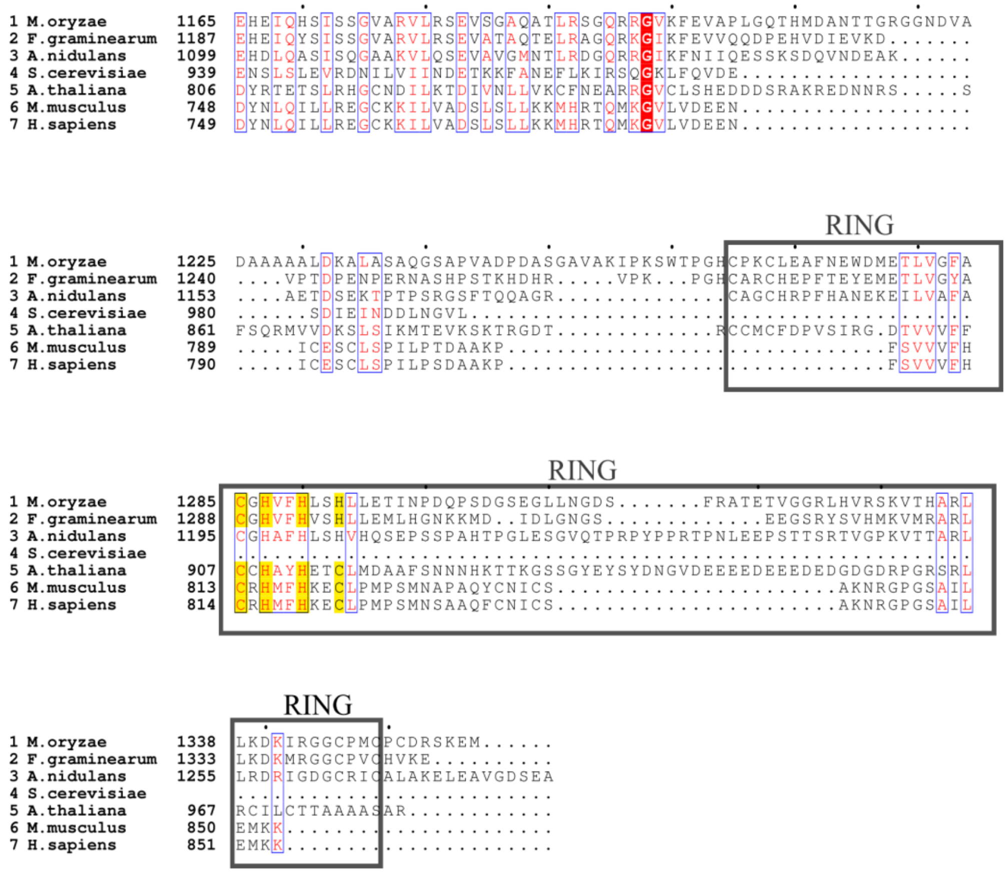

Supplementary figure 2. Amino acid alignment of MoVps41 and its homologues with other vacuolar protein sorting-associated proteins. The amino acid alignment of the Vps of *M. oryzae*, *S. cerevisiae*, *F. graminearum* , *A. thaliana* , *M. musculus* , and *H. Sapiens*, was performed using ClustalW. All these proteins possess the six conserved clathrin domains of vacuolar protein sorting. A comparison of the RING finger motif H2 variant of MoVps41 with that of *F.graminearum* , *A.thaliana* , *M. musculus* , and *H. Sapiens*. The conserved cysteine and histidine residues of these motifs have been highlighted, and the consensus sequence is shown.
